# A panoramic continuous compressive beamformer with cuboid microphone arrays

**DOI:** 10.1038/s41598-019-47845-7

**Published:** 2019-08-19

**Authors:** Yang Yang, Zhigang Chu, Yong-Xin Yang, Zhongming Xu, Yongxiang Zhang

**Affiliations:** 1 Faculty of Vehicle Engineering, Chongqing Industry Polytechnic College, Chongqing, 401120 China; 2 State Key Laboratory of Mechanical Transmissions, Chongqing University, Chongqing, 400044 China

**Keywords:** Acoustics, Mechanical engineering, Electrical and electronic engineering

## Abstract

Compressive beamforming is a powerful approach for the direction-of-arrival (DOA) estimation and strength quantification of acoustic sources. The conventional grid-based discrete compressive beamformer suffers from the basis mismatch conundrum. Its result degrades under the situation that sources fall off the grid. The existing continuous compressive beamformer with linear or planar microphone arrays can circumvent the conundrum, but work well only for sources in a local region. Here we develop a panoramic continuous compressive beamformer with cuboid microphone arrays based on an atomic norm minimization (ANM) and a matrix pencil and paring method. To solve the positive semidefinite programming equivalent to the ANM efficiently, we formulate a solving algorithm based on the alternating direction method of multipliers. We also present an iterative reweighted ANM to enhance sparsity and resolution. The beamformer is capable of estimating the DOAs and quantifying the strengths of acoustic sources panoramically and accurately, whether a standard uniform or a sparse cuboid microphone array is utilized.

## Introduction

Compressive sensing^1–3^ based beamforming is an emerging and powerful approach for the direction-of-arrival (DOA) estimation and strength quantification of acoustic sources, which is also simply called compressive beamforming^4,5^. It measures signals with an array of microphones, and then processes the signals to retrieve the direction and strength information of sources by exploiting the source sparsity. Numerous applications can be encountered, for example, noise source identification in environmental protection, target detection in military surveillance, fault diagnosis in equipment maintenance, speaker tracking in video conference, etc.

In conventional compressive beamformer, the DOA domain is gridded/discretized into a finite set of look directions and all the sources are assumed to fall in these look directions. An underdetermined linear system of equations is established relating the signals measured by microphones to the unknown source distribution. It is solved by imposing a sparsity constraint. Under a single snapshot, that is minimizing the ${\ell }_{{\rm{1}}}$ norm of the vector composed by the source strengths in all the look directions. Under the multiple snapshots, the ${\ell }_{{\rm{1}}}$ norm of the vector becomes the ${\ell }_{2,1}$ norm of a matrix. Its results become inaccurate when the DOAs of sources do not conform with these look directions. The problem is termed as basis mismatch^4,6^ and can be often encountered in practical applications. Using finer grids mitigates the problem but increases the complexity of computation. More seriously, grid refinement makes the coherence of the measuring process increase, which can cause offset in the estimates^4^.

Inspired by the continuous methods in the field of frequency retrieval^7–14^, some scholars have recently developed the continuous compressive beamformer for the DOA estimation and strength quantification of acoustic sources. Based on the minimization of the atomic norm of source strength and the polynomial rooting method, Xenaki *et al*.^15^ developed a one-dimensional single-snapshot continuous compressive beamformer for the measurement with linear microphone arrays. Park *et al*.^16^ extended Xenaki *et al*.’s beamformer to the multiple-snapshot case via the group atomic norm. The authors^17–20^ developed a two-dimensional single-snapshot continuous compressive beamformer for the measurement with rectangular microphone arrays based on the minimization of the atomic norm of microphone signal induced by sources and the matrix enhancement and matrix pencil method^21^, and extended it to the multiple-snapshot case via the group atomic norm and the matrix pencil and pairing (MaPP) method^13,14^. Treating the DOAs of sources as a continuum, these beamformer can sidestep the basis mismatch conundrum fundamentally. Moreover, the multiple-snapshot data help to obtain more accurate and robust results compared with the single-snapshot ones^16,20^. In the linear array, there are only a line of microphones. In the rectangular array, there are only a plane of microphones. When two sources distribute symmetrically about the line or the plane, they induce the same signals at each microphone, and thus will not be distinguished. Consequently, the measurement with linear microphone arrays requires that the sources must be in front of the array and coplanar with the microphones. The measurement with rectangular microphone arrays requires that the sources must be in front of the array. That means the existing continuous compressive beamformer cannot yet panoramically estimate the DOAs and quantify the strengths of acoustic sources. This paper is dedicated to realizing this function with a cuboid microphone array. It is impossible for two sources to induce the same signals at each microphone in a cuboid microphone array. Therefore, the panoramic estimation will be allowed. The work is of great significance because the sources scatter throughout the whole three-dimensional space in a mass of practical situations.

The key contributions are as follows: (1) we develop a panoramic continuous compressive beamformer with cuboid microphone arrays under the multiple-snapshot data model. Four steps are involved. First, an atomic norm minimization (ANM) is defined to denoise the measured signal and get the microphone signal induced by sources. Then, a positive semidefinite programming equivalent to the ANM is formulated and solved. Subsequently, the MaPP method is utilized to process the result of the positive semidefinite programming and estimate DOAs. Finally, the source strengths are quantified based on the estimated DOAs and the obtained microphone signal from sources. (2) Based on alternating direction method of multipliers (ADMM)^22–24^, we formulate a reasonably fast algorithm to solve the positive semidefinite programming. (3) We enhance sparsity and resolution via an iterative reweighted ANM (IRANM). (4) We investigate the applicability of the beamformer to the sparse cuboid microphone arrays, which are constructed via randomly retaining microphones from the standard uniform cuboid microphone arrays.

This paper is directly inspired by refs^13,14^, where a multidimensional super-resolution frequency retrieval approach was proposed. The main connections and differences between them are as follows: (1) refs^13,14^ focus on the problem of frequency retrieval, whereas this paper solves the problem of DOA estimation and source strength quantification. (2) The ANM and IRANM in this paper are enlightened by the convex relaxation method and the reweighted trace minimization method in refs^13,14^. Differently, the methods in refs^13,14^ are based on the single-snapshot data model, whereas the methods in this paper are based on the multiple-snapshot one. As proved by the literatures in one- and two-dimensional fields^5,10,12,16,20^, the multiple-snapshot method performs better. (3) In this paper, we deduce and develop an algorithm based on ADMM to solve the positive semidefinite programming, and demonstrate its advantage over the IPM based SDPT3 solver. References^13,14^ did not do this work. (4) Reference^14^ only introduces the concept of permutation matrices abstractly when describing the MaPP method, whereas this paper presents the concrete expressions. (5) In this paper, we investigate the effect rule of the estimated value of noise level on the results and give out advices. References^13,14^ did not do this work.

## Results

### Accurate DOA estimation and strength quantification function of the panoramic continuous compressive beamformer

**Figure 1:**
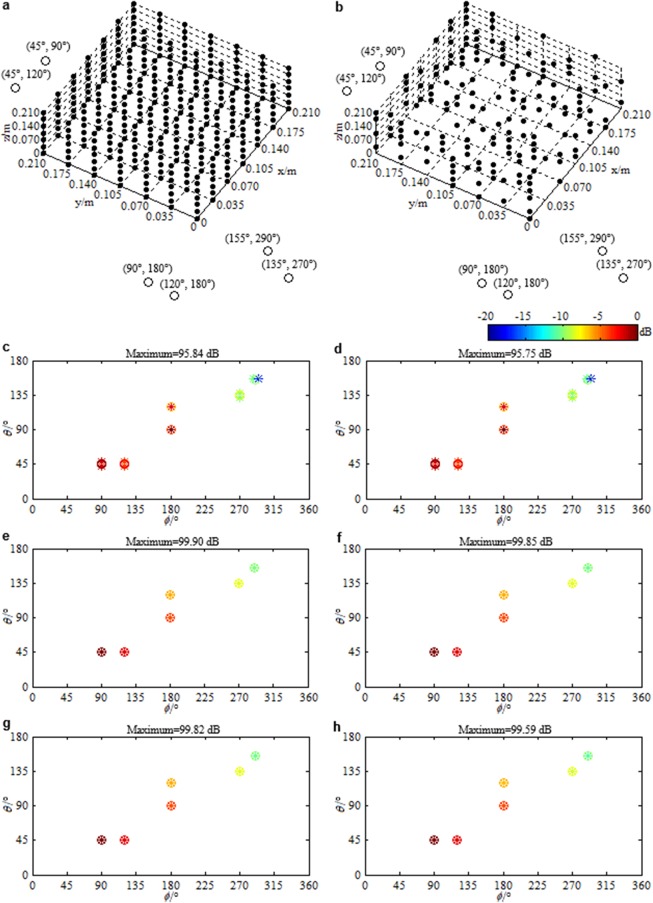
Microphone arrays and reconstructed source distributions. (**a**) The standard uniform cuboid microphone array for (**c**,**e** and **g**). (**b**) The sparse cuboid microphone array for (**d**,**f** and **h**). ● Represents the microphone and ○ represents the source. Source distributions reconstructed by (**c**,**d**) the conventional and (**e**–**h**) the developed panoramic continuous compressive beamformer. The positive semidefinite programming equivalent to the ANM is solved by (**e**,**f**) the SDPT3 solver in CVX toolbox and (**g**,**h**) our ADMM based algorithm. In (**c**–**h**), the reconstructed (*) and the true (○) outputs are scaled to dB via referring to their respective maximum, and at the same time, referring to 2 × 10^−5^ Pa, the reconstructed maximum is labeled on the top, so do in the subsequent maps.

**Figure 2:**
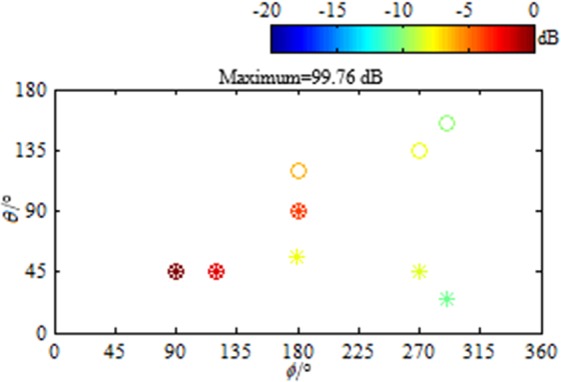
Source distribution reconstructed by two-dimensional continuous compressive beamformer.

Here, we show the function of the developed beamformer via a simulation example. Assume six sources. Their DOAs, expressed by (*θ*, *ϕ*) with *θ* ∈ [0°, 180°] being the elevation angle and *ϕ* ∈ [0°, 360°] being the azimuth angle, are (45°, 90°), (45°, 120°), (90°, 180°), (120°, 180°), (135°, 270°) and (155°, 290°), in turn. Their root mean square strengths, expressed by ${s}_{rms}={\Vert {\bf{s}}\Vert }_{2}/\sqrt{L}$ with ${\bf{s}}\in {{\rm{C}}}^{1\times L}$ being the row vector composed by the source strength under each snapshot, C being the set of complex numbers, *L* being the total number of snapshots and ||·||_2_ being the ${\ell }_{2}$ norm, are 100 dB, 98 dB, 96 dB, 94 dB, 92 dB and 90 dB (referring to 2 × 10^−5^ Pa). The frequency of emitted signal is 4000 Hz. Figure 1 presents the utilized microphone arrays and the reconstructed source distributions. A standard uniform cuboid array with 343 microphones (Fig. 1a) is utilized to obtain Fig. 1c,e,g, while a sparse cuboid array with 170 microphones (Fig. 1b) is utilized to obtain Fig. 1d,f,h. Figure 1a,b also presents the panoramic scene of sources. Figure 1c,d corresponds to the conventional compressive beamformer. The grid is [0°: 6°: 180°] × [0°: 6°: 360°]. Obviously, only the third and fourth sources that lie on the grid are accurately identified. For the other sources that lie off the grid, leakage occurs, which leads to inaccurate DOA estimations and peaks seriously deviating from the true source strengths. In contrast, as shown in Fig. 1e–h, the developed beamformer estimates the DOA and quantifies the strength of each source accurately. To sum up, the developed panoramic continuous compressive beamformer can conquer the basis mismatch conundrum, allowing accurate DOA estimation and strength quantification, whether a standard uniform or a sparse cuboid microphone array is utilized. Besides, Fig. 2 presents the result reconstructed by the two-dimensional continuous compressive beamformer with a rectangular microphone array. Only sources whose elevation angels lie in [0°, 90°] are accurately identified. This demonstrates the necessity of developing the panoramic continuous compressive beamformer to realize the panoramic DOA estimation and strength quantification of acoustic sources. To obtain Figs 1 and 2, multiple snapshots are adopted. An example is also exhibited in Supplementary Note 1 to again demonstrate the advantages of the multiple-snapshot data model over the single-snapshot one, which has been demonstrated and explained in one- and two-dimensional fields^5,10,12,16,20^.

### Efficiency advantage of our ADMM based algorithm

To obtain Fig. 1e,f, we solve the positive semidefinite programming equivalent to the ANM by the off-the-peg SDPT3 solver in CVX toolbox^25^, which uses the interior point method (IPM)^26^. To obtain Fig. 1g,h, we solve the positive semidefinite programming by our ADMM based algorithm. The accurate DOA estimation and strength quantification shown in Fig. 1e–h demonstrates that both the solver and our algorithm are effective. Employ ${\Vert \hat{{\bf{P}}}-{\bf{P}}\Vert }_{{\rm{F}}}/{\Vert {\bf{P}}\Vert }_{{\rm{F}}}$, ${\Vert [\hat{{\boldsymbol{\theta }}},\hat{{\boldsymbol{\varphi }}}]-[{\rm{\theta }},{\boldsymbol{\varphi }}]\Vert }_{{\rm{F}}}/2I$ and ${\Vert {\hat{{\bf{s}}}}_{rms}-{{\bf{s}}}_{rms}\Vert }_{2}/{\Vert {{\bf{s}}}_{rms}\Vert }_{2}$ to measure the microphone signal reconstruction error, the DOA estimation error and the strength quantification error. Thereinto, ||·|||_F_ is the Frobenius norm, $\hat{{\bf{P}}}\in {{\rm{C}}}^{ABC\times L}$ and ${\bf{P}}\in {{\rm{C}}}^{ABC\times L}$ are the matrices of the reconstructed and the true microphone signals respectively, *A*, *B* and *C* are the row, column and layer numbers of the cuboid microphone array, *ABC* is the total number of microphones, $\hat{{\boldsymbol{\theta }}}\in {{\rm{R}}}^{I}$ and ${\boldsymbol{\theta }}\in {{\rm{R}}}^{I}$ are the vectors of the estimated and the true elevation angles respectively, R is the set of real numbers, *I* is the total number of sources, $\hat{{\boldsymbol{\varphi }}}\in {{\rm{R}}}^{I}$ and ${\boldsymbol{\varphi }}\in {{\rm{R}}}^{I}$ are the vectors of the estimated and the true azimuth angles respectively, and ${\hat{{\bf{s}}}}_{rms}\in {{\rm{R}}}^{I}$ and **s**_*rms*_ ∈ R^*I*^ are the vectors of the quantified and the true root mean square strengths respectively. Table 1 lists the values of these errors corresponding to Fig. 1e–h and the consuming time of the IPM based SDPT3 solver and our ADMM based algorithm. Apparently, in terms of microphone signal reconstruction and strength quantification, the panoramic continuous compressive beamformer with the ADMM based algorithm has slightly lower accuracy than the one with the IPM based SDPT3 solver, whereas in term of DOA estimation, they have almost the same accuracy. The consuming time of our ADMM based algorithm is only about 1/7 of the one of the IPM based SDPT3 solver at most. Besides, when the dimensionality of the problem increases, for example, *A* = *B* = *C* = 8, the IPM based SDPT3 solver fails to obtain effective solutions, whereas our ADMM based algorithm still works well.

**Figure 3:**
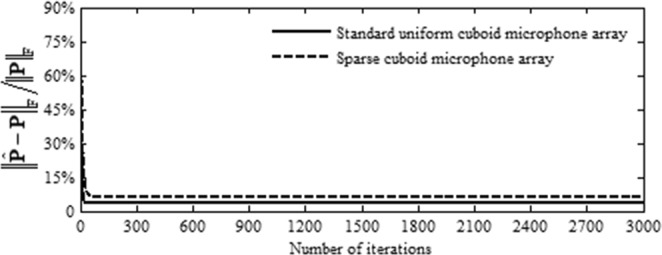
Curves of ${\Vert \hat{{\bf{P}}}-{\bf{P}}\Vert }_{{\rm{F}}}/{\Vert {\bf{P}}\Vert }_{{\rm{F}}}$ vs. number of iterations in our ADMM based algorithm.

To test the convergence of our ADMM based algorithm, we plot the curves of the error ${\Vert \hat{{\bf{P}}}-{\bf{P}}\Vert }_{{\rm{F}}}/{\Vert {\bf{P}}\Vert }_{{\rm{F}}}$ vs. the number of iterations in Fig. 3. The parameter setup of sources keeps the same as in Fig. 1. Obviously, whether the standard uniform or the sparse cuboid microphone array is utilized, a plateau is reached after only a few dozens of iterations, which means our algorithm can converge fast. The errors after convergence are 3.38% and 6.38% respectively for the standard uniform cuboid microphone array and the sparse one, which are larger than the errors of the IPM based SDPT3 solver (2.45% and 3.32%). Changing the parameters, such as number and DOAs of sources, frequency, SNR and so on, to conduct simulations, similar phenomena can be obtained. See Supplementary Note 2. These phenomena demonstrate the inherent characteristic of ADMM that it converges fast to a moderate accuracy, but slowly even impossibly to an extremely accurate solution^11,22^. Fortunately, the moderate accuracy is typically sufficient in practical applications.

### Sparsity and resolution enhancement via IRANM in the case of small source separation

**Figure 4:**
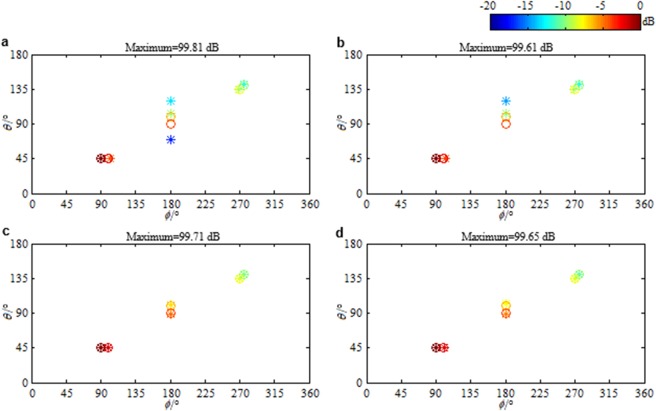
Reconstructed source distributions. (**a**,**b**) The ANM and (**c**,**d**) the IRANM based panoramic continuous compressive beamformer are utilized. (**a**,**c**) The standard uniform and (**b**,**d**) the sparse cuboid microphone array are utilized.

Define the minimum separation among sources as

1 $${{\rm{\Delta }}}_{{\rm{\min }}}=\mathop{{\rm{\min }}}\limits_{\begin{array}{c}i,i^{\prime} \in \{1,2,\cdots ,I\}\\ i\ne i^{\prime} \end{array}}\,{\rm{\max }}\{|{t}_{1i}-{t}_{1i^{\prime} }|,|{t}_{2i}-{t}_{2i^{\prime} }|,|{t}_{3i}-{t}_{3i^{\prime} }|\},$$

where both *i* and *i*′ are the source indices, and *t*_1*i*_(*t*_1*i*′_), *t*_2*i*_(*t*_2*i*′_) and *t*_3*i*_(*t*_3*i*′_) are the projections of the DOA of the *i*th (*i*′th) source on the *x*, *y* and *z* dimensions of the microphone array respectively. Denote by *θ*_*i*_ and ${\varphi }_{i}$ the elevation and the azimuth angle of the *i*th source, Δ*x*, Δ*y* and Δ*z* the microphone spaces, and *λ* the wavelength, then ${t}_{1i}\equiv \,\sin \,{\theta }_{i}\,\cos \,{\varphi }_{i}{\rm{\Delta }}x/\lambda$, ${t}_{2i}\equiv \,\sin \,{\theta }_{i}\,\sin \,{\varphi }_{i}{\rm{\Delta }}y/\lambda$ and ${t}_{3i}\equiv \,\cos \,{\theta }_{i}{\rm{\Delta }}z/\lambda$. A smaller minimum separation among sources that can be accurately identified means a higher resolution. Change the DOAs of the second, fourth and sixth sources assumed in Fig. 1 as (45°, 100°), (100°, 180°) and (140°, 275°) in turn. Namely, let the first and second sources, the third and fourth sources, and the fifth and sixth sources close to each other. Δ_min_ decreases from 0.13 to 0.03. Figure 4 presents the reconstructed source distributions. The standard uniform cuboid array with 343 microphones (Fig. 1a) is utilized to obtain Fig. 4a,c, while the sparse cuboid array with 170 microphones (Fig. 1b) is utilized to obtain Fig. 4b,d. Figure 4a,b corresponds to the ANM based panoramic continuous compressive beamformer. It fails to identify all the sources accurately. The estimated number of sources in Fig. 4a is also more than the true one. In contrast, as shown in Fig. 4c,d, the IRANM based panoramic continuous compressive beamformer estimates the DOA and quantifies the strength of each source accurately. The phenomenon demonstrates that IRANM can enhance sparsity and resolution, whether a standard uniform or a sparse cuboid microphone array is utilized.

**Figure 5:**
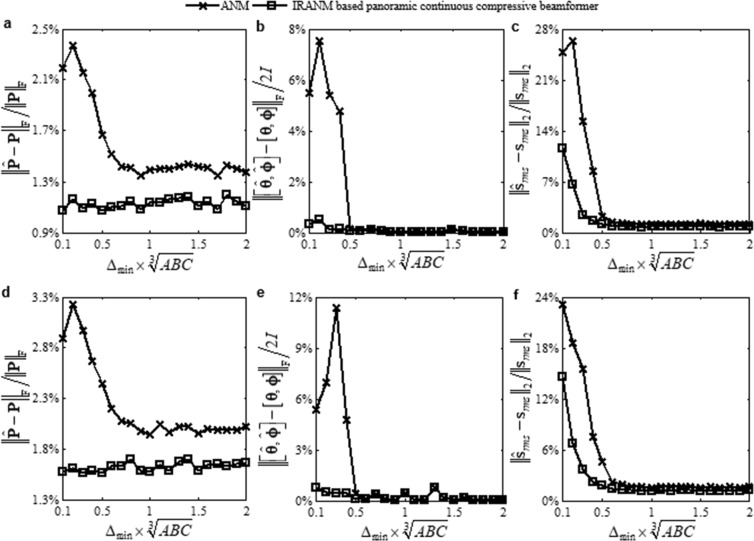
Error curves. (**a**,**d**) ${\Vert \hat{{\bf{P}}}-{\bf{P}}\Vert }_{{\rm{F}}}/{\Vert {\bf{P}}\Vert }_{{\rm{F}}}$, (**b**,**e**) ${\Vert [\hat{{\boldsymbol{\theta }}},\hat{{\boldsymbol{\varphi }}}]-[{\boldsymbol{\theta }},{\boldsymbol{\varphi }}]\Vert }_{{\rm{F}}}/2I$ and (**c**,**f**) ${\Vert {\hat{{\bf{s}}}}_{rms}-{{\bf{s}}}_{rms}\Vert }_{2}/{\Vert {{\bf{s}}}_{rms}\Vert }_{2}$ vs. Δ_min_. (**a**–**c**) The standard uniform and (**d**–**f**) the sparse cuboid microphone array are utilized.

Figure 5 plots the curves of the above errors vs. Δ_min_ of the ANM and the IRANM based panoramic continuous compressive beamformer. For each Δ_min_, the errors are measured over 20 Monte Carlo runs. Two sources that are separated by Δ_min_ are generated in each run. The standard uniform cuboid array with 343 microphones is utilized to obtain Fig. 5a–c, while a sparse cuboid array with only 170 randomly retained microphones is utilized to obtain Fig. 5d–f. In term of reconstruction of **P**, Fig. 5a,d shows that the error of ANM is distinctly higher under ${{\rm{\Delta }}}_{{\rm{\min }}} < 0.7/\sqrt[{\rm{3}}]{ABC}$ compared to ${{\rm{\Delta }}}_{{\rm{\min }}}\ge 0.7/\sqrt[{\rm{3}}]{ABC}$, whereas the error of IRANM varies only slightly across all Δ_min_ and is distinctly lower than the one of ANM. In term of DOA estimation, Fig. 5b,e shows that on one hand, the IRANM based panoramic continuous compressive beamformer has very low errors across all Δ_min_; on the other hand, compared to the former, the ANM based panoramic continuous compressive beamformer has distinctly higher errors under ${{\rm{\Delta }}}_{{\rm{\min }}} < 0.5/\sqrt[{\rm{3}}]{ABC}$ and almost the same errors under ${{\rm{\Delta }}}_{{\rm{\min }}}\ge 0.5/\sqrt[{\rm{3}}]{ABC}$. In term of strength quantification, Fig. 5c,f shows that the error of the ANM based panoramic continuous compressive beamformer is always higher than the one of the IRANM based panoramic continuous compressive beamformer, and that is particularly apparent under small Δ_min_. These phenomena demonstrate that the IRANM based panoramic continuous compressive beamformer has the stronger denoising capability and the enhanced resolution compared to the ANM based one, allowing to reconstruct the microphone signal induced by sources as well as estimate the DOAs and quantify the strengths of small-separation sources more accurately, whether a standard uniform or a sparse cuboid microphone array is utilized.

### Sparsity and resolution enhancement via IRANM in the case of underestimated noise level

**Figure 6:**
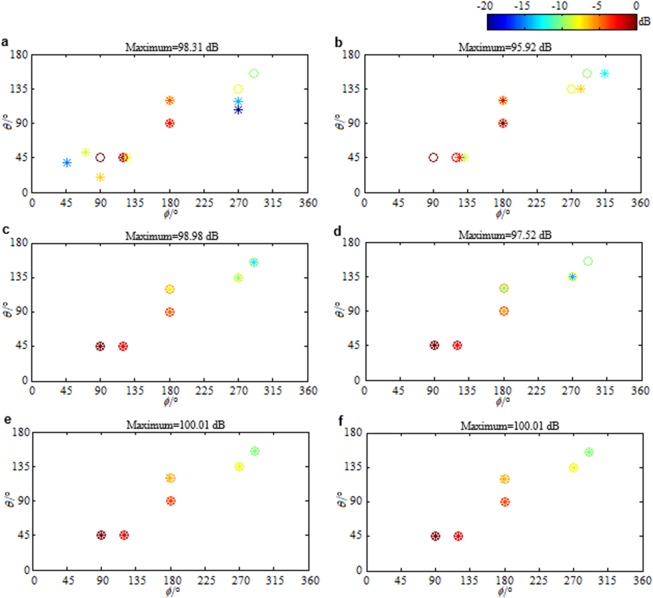
Reconstructed source distributions. (**a**–**d**) The ANM and (**e**,**f**) the IRANM based panoramic continuous compressive beamformer are utilized. *ε* is (**a**,**e**) $0.25{\Vert {\bf{N}}\Vert }_{{\rm{F}}}$, (**b**,**f**) $0.5{\Vert {\bf{N}}\Vert }_{{\rm{F}}}$, (**c**) $2{\Vert {\bf{N}}\Vert }_{{\rm{F}}}$ and (**d**) $4{\Vert {\bf{N}}\Vert }_{{\rm{F}}}$. The standard uniform cuboid microphone array is utilized.

**Figure 7:**
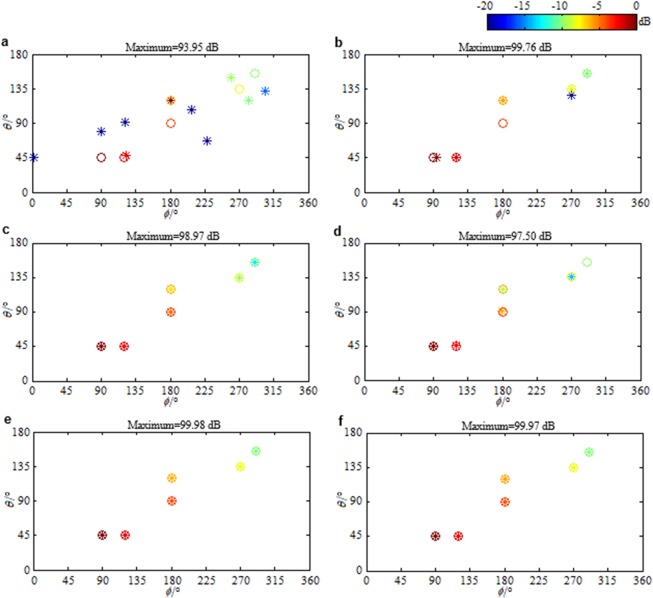
Reconstructed source distributions. (**a**–**d**) The ANM and (**e**,**f**) the IRANM based panoramic continuous compressive beamformer are utilized. *ε* is (**a**,**e**) $0.25{\Vert {\bf{N}}\Vert }_{{\rm{F}}}$, (**b**,**f**) $0.5{\Vert {\bf{N}}\Vert }_{{\rm{F}}}$, (**c**) $2{\Vert {\bf{N}}\Vert }_{{\rm{F}}}$ and (**d**) $4{\Vert {\bf{N}}\Vert }_{{\rm{F}}}$. The sparse cuboid microphone array is utilized.

In above results, the noise level is estimated accurately. Namely, the estimated noise level *ε* is equal to the true one (the Frobenius norm of the true noise ${\bf{N}}\in {{\rm{C}}}^{ABC\times L}$). In practical applications, the noise is usually not explicitly known, and therefore, it is hard to let *ε* be equal to the true noise level. Figure 6a–d respectively presents the distributions reconstructed by the ANM based panoramic continuous compressive beamformer for the sources assumed in Fig. 1 when *ε* is $0.25{\Vert {\bf{N}}\Vert }_{{\rm{F}}}$, $0.5{\Vert {\bf{N}}\Vert }_{{\rm{F}}}$, $2{\Vert {\bf{N}}\Vert }_{{\rm{F}}}$ and $4{\Vert {\bf{N}}\Vert }_{{\rm{F}}}$. In Fig. 6a, the estimated number of sources is more than the true one, and the DOA estimation as well as the strength quantification is wrong. In Fig. 6b, even though the estimated sources in the display dynamic range have the same number as the true ones, the DOA estimation as well as the strength quantification is still wrong. In Fig. 6c, the DOAs are accurately estimated. The source strengths are quantified as 98.98 dB, 96.71 dB, 94.22 dB, 91.71 dB, 89.11 dB and 86.17 dB in turn, which are 1.02 dB, 1.29 dB, 1.78 dB, 2.29 dB, 2.89 dB and 3.83 dB lower than the true values. Distinctly, the weaker the source is, the more the quantified strength is lower than the true one. In Fig. 6d, the weakest source in (155°, 290°) has been lost. These phenomena demonstrate that for the ANM based panoramic continuous compressive beamformer, an underestimated noise level will lead to a less sparse solution and thus a wrong identification and a reduced resolution, whereas an overestimated noise level will cause a too sparse solution by eliminating the weak sources. This is mainly because by underestimating noise level, partial noise is identified as sources, whereas by overestimating noise level, the weak sources are removed as noise. Figure 6e,f presents the source distributions reconstructed by the IRANM based panoramic continuous compressive beamformer when *ε* is $0.25{\Vert {\bf{N}}\Vert }_{{\rm{F}}}$ and $0.5{\Vert {\bf{N}}\Vert }_{{\rm{F}}}$. Apparently, all the DOAs are estimated and all the strengths are quantified accurately, which means IRANM enhances the sparsity and resolution. The standard uniform cuboid array with 343 microphones is utilized to obtain Fig. 6. Figure 7 presents the results when the sparse cuboid array with 170 microphones is utilized. It has the same rule as Fig. 6.

## Discussion

In summary, we develop a panoramic continuous compressive beamformer with cuboid microphone arrays. It first denoises the measured signal and thus obtains the signal from sources by minimizing a sparse metric of source distribution in the continuous domain, for example, the atomic norm of the microphone signal induced by sources, then retrieves the DOA information via using the MaPP method to process the result of ANM, and finally quantifies the source strength according to the DOA information and the microphone signal from sources. The beamformer can conquer the basis mismatch conundrum of the conventional grid-based discrete compressive beamformer, allowing panoramic and accurate DOA estimation and strength quantification of acoustic sources, whether a standard uniform or a sparse cuboid microphone array is utilized.

To solve the ANM, we formulate a positive semidefinite programming. It is a convex optimization problem and can be solved by the off-the-peg IPM based SDPT3 solver in CVX toolbox. The solver trends to be slow and even ineffective for large-dimensionality problems. To overcome the limitation, we present a reasonably fast algorithm based on ADMM. By establishing another sparse metric, we also present the IRANM that can be substituted for ANM. Because the metric promotes the sparsity to a larger degree than the atomic norm, IRANM can enhance sparsity and resolution compared with ANM. For the sources with a small separation, the ANM based beamformer fails to estimate the DOAs and quantify the strengths accurately, whereas the IRANM based beamformer succeeds. The beamformer takes the estimated noise level as an input. For the ANM based beamformer, overestimating the noise level may make the solution too sparse, for instance by eliminating sources of smaller strength. On the other hand, by underestimating the noise level, the solution may be less sparse than the actual solution, which will leads to inaccurate identifications and reduced resolution. In practical applications, when the noise is not explicitly known, in order to capture all the sources, we advise using a properly underestimated noise level and enhancing sparsity and resolution by IRANM.

The IRANM is solved only by the slow SDPT3 solver in this paper. We have not yet successfully developed a fast algorithm for it, even based on ADMM. The reason why IRANM cannot be solved accurately and robustly by ADMM can be explained as follows. IRANM is an iterative strategy. The weighting matrix utilized in current iteration is calculated based on the results in the previous iteration. In each iteration, if the semidefinite positive programming is solved via ADMM, the inherent characteristic that ADMM cannot fast converge to an accurate solution^11,22^ will lead to a result with moderate accuracy. The error will affect the weighting matrix and thus burden the result with a larger error in next iteration. Consequently, as the iteration increases, the actually solved result deviates from the theoretical one more and more. In the future work, it is of significance to handle the problem. Besides, no experimental data are provided to support the claims in this paper. The accuracy of the method becomes lower as the SNR decreases. It is also of significance to conduct the experimental investigation and explore a new method that can still enjoy high accuracy even under very low, for example, negative, SNRs. Finally, it is also of interest to develop the panoramic continuous compressive beamformer with other microphone arrays, for example, spherical ones, to realize the panoramic and accurate DOA estimation and source strength quantification.

## Methods

### Problem formulation

**Figure 8:**
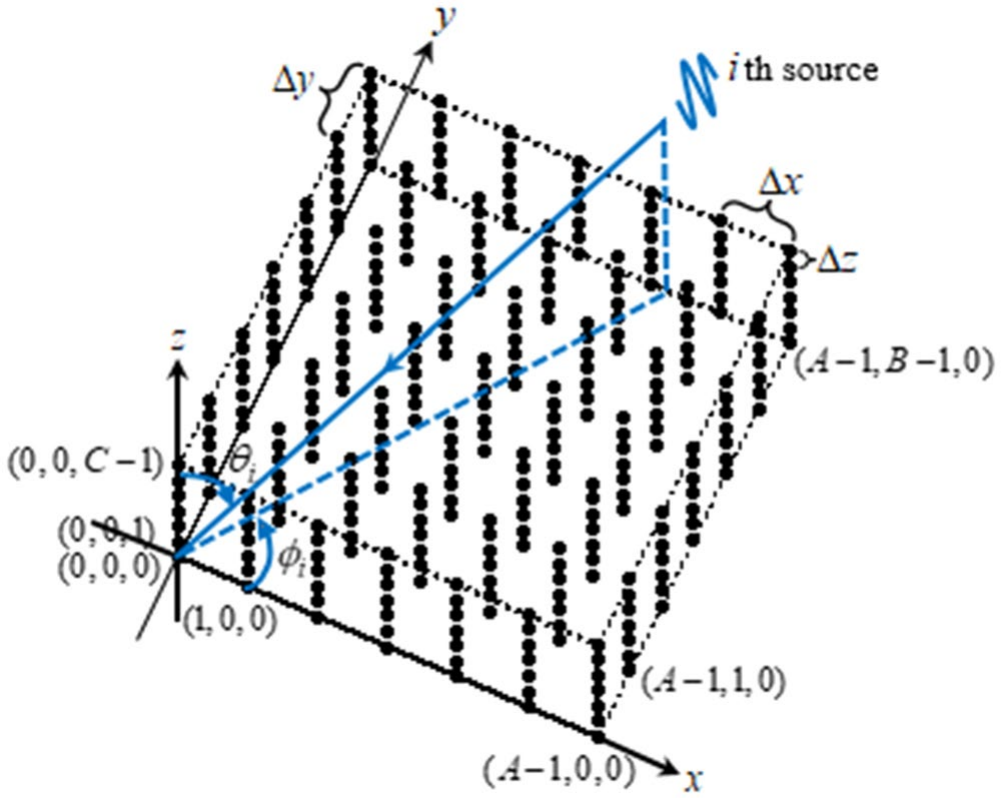
Measurement layout.

Figure 8 depicts the measurement layout with a cuboid microphone array. (*a*, *b*, *c*) with *a* = 0, 1, …, *A* − 1, *b* = 0, 1, …, *B* − 1 and *c* = 0, 1, …, *C* − 1 indexes the microphone. (*θ*_*i*_, *ϕ*_*i*_) expresses the DOA of the *i*th source. Under the assumption of plane wave, the row vector ${{\bf{p}}}_{a,b,c}\in {{\rm{C}}}^{1\times L}$ of the signal induced by sources at (*a*, *b*, *c*)th microphone under each snapshot can be modeled as

2 $${{\bf{p}}}_{a,b,c}=\mathop{\sum }\limits_{i=1}^{I}{{\bf{s}}}_{i}{e}^{j2\pi ({t}_{1i}a+{t}_{2i}b+{t}_{3i}c)},$$

where $j=\sqrt{-1}$ is the imaginary unit, ${{\bf{s}}}_{i}=[{s}_{i,1},{s}_{i,2},\cdots ,{s}_{i,L}]\in {{\rm{C}}}^{1\times L}$, ${s}_{i,l}$ expresses the strength of the *i*th source under the *l*th snapshot, which is the signal induced by the source at the (0, 0, 0)th microphone.

After forming the matrix $${\bf{P}}=[{{\bf{p}}}_{0,0,0}^{{\rm{T}}},{{\bf{p}}}_{0,0,1}^{{\rm{T}}}$$, …, $${{\bf{p}}}_{0,0,C-1}^{{\rm{T}}},{{\bf{p}}}_{0,1,0}^{{\rm{T}}},{{\bf{p}}}_{0,1,1}^{{\rm{T}}}$$, …, $${{\bf{p}}}_{0,1,C-1}^{{\rm{T}}}$$, …, $${{\bf{p}}}_{0,B-1,0}^{{\rm{T}}},{{\bf{p}}}_{0,B-1,1}^{{\rm{T}}}$$, …, $${{\bf{p}}}_{0,B-1,C-1}^{{\rm{T}}},{{\bf{p}}}_{1,0,0}^{{\rm{T}}},{{\bf{p}}}_{1,0,1}^{{\rm{T}}}$$, …, $${{\bf{p}}}_{1,0,C-1}^{{\rm{T}}},{{\bf{p}}}_{1,1,0}^{{\rm{T}}},{{\bf{p}}}_{1,1,1}^{{\rm{T}}}$$, …, $${{\bf{p}}}_{1,1,C-1}^{{\rm{T}}}$$, …, $${{\bf{p}}}_{1,B-1,0}^{{\rm{T}}},{{\bf{p}}}_{1,B-1,1}^{{\rm{T}}}$$, …, $${{\bf{p}}}_{1,B-1,C-1}^{{\rm{T}}}$$, …, $${{\bf{p}}}_{A-1,0,0}^{{\rm{T}}},{{\bf{p}}}_{A-1,0,1}^{{\rm{T}}}$$, …, $${{\bf{p}}}_{A-1,0,C-1}^{{\rm{T}}},{{\bf{p}}}_{A-1,1,0}^{{\rm{T}}},{{\bf{p}}}_{A-1,1,1}^{{\rm{T}}}$$, …, $${{\bf{p}}}_{A-1,1,C-1}^{{\rm{T}}}$$, …, $${{\bf{p}}}_{A-1,B-1,0}^{{\rm{T}}},{{\bf{p}}}_{A-1,B-1,1}^{{\rm{T}}}$$, …, $${{\bf{p}}}_{A-1,B-1,C-1}^{{\rm{T}}}{]}^{{\rm{T}}}\in {{\rm{C}}}^{ABC\times L}$$ and the column vector $${\bf{d}}({t}_{1i},{t}_{2i},{t}_{3i})={[1,{e}^{j2\pi {t}_{1i}},\cdots ,{e}^{j2\pi {t}_{1i}(A-1)}]}^{{\rm{T}}}$$ ⊗ [$$1,{e}^{j2\pi {t}_{2i}},\cdots ,$$$${e}^{j2\pi {t}_{2i}(B-1)}$$]^T^ ⊗ $${[1,{e}^{j2\pi {t}_{3i}},\cdots ,{e}^{j2\pi {t}_{3i}(C-1)}]}^{{\rm{T}}}\in {{\rm{C}}}^{ABC}$$, where (·)^T^ and ⊗ denote the transpose and the Kronecker product operator respectively, we obtain

3 $${\bf{P}}=\mathop{\sum }\limits_{i=1}^{I}{\bf{d}}({t}_{1i},{t}_{2i},{t}_{3i}){{\bf{s}}}_{i}.$$

Denoting by ${\bf{N}}\in {{\rm{C}}}^{ABC\times L}$ the additive noise, the measurement matrix ${{\bf{P}}}^{\bigstar}\in {{\rm{C}}}^{ABC\times L}$ is described by

4 $${{\bf{P}}}^{\bigstar}={\bf{P}}+{\bf{N}}.$$

The primary focus of this paper is to retrieve the DOA and strength information of sources with **P**^⭑^ and the estimated noise level as inputs and without discretizing the target region into a grid. Equation (4) becomes ${{\bf{P}}}_{{\rm{\Omega }}}^{\bigstar}={{\bf{P}}}_{{\rm{\Omega }}}+{{\bf{N}}}_{{\rm{\Omega }}}$ for sparse cuboid microphone arrays, where Ω denotes the set of the indices of the retained microphones, ${{\bf{P}}}_{{\rm{\Omega }}}^{\bigstar}\in {{\rm{C}}}^{|{\rm{\Omega }}|\times L}$ denotes the matrix of the signals measured by the retained microphones, ${{\bf{P}}}_{{\rm{\Omega }}}\in {{\rm{C}}}^{|{\rm{\Omega }}|\times L}$ denotes the matrix of the signals induced by sources at the retained microphones, ${{\bf{N}}}_{{\rm{\Omega }}}\in {{\rm{C}}}^{|{\rm{\Omega }}|\times L}$ denotes the matrix of the noises born by the retained microphones, and |Ω| denotes the cardinality of Ω. This paper also focuses on the applicability of the developed beamformer to the sparse cuboid microphone arrays.

### ANM

Let ${s}_{i}={\Vert {{\bf{s}}}_{i}\Vert }_{2}\in {{\rm{R}}}^{+}$ and ${{\boldsymbol{\psi }}}_{i}={{\bf{s}}}_{i}/{s}_{i}\in {{\rm{C}}}^{1\times L}$, where R^+^ is the set of positive real numbers and ${\Vert {{\boldsymbol{\psi }}}_{i}\Vert }_{2}=1$. We reformulate Eq. (3) as

5 $${\bf{P}}=\mathop{\sum }\limits_{i=1}^{I}{s}_{i}{\bf{d}}({t}_{1i},{t}_{2i},{t}_{3i}){{\boldsymbol{\psi }}}_{i}.$$

Under the continuous setting, ${t}_{1}\equiv \,\sin \,\theta \,\cos \,\varphi {\rm{\Delta }}x/\lambda$, ${t}_{2}\equiv \,\sin \,\theta \,\sin \,\varphi {\rm{\Delta }}y/\lambda$, ${t}_{3}\equiv \,\cos \,\theta {\rm{\Delta }}z/\lambda$ and the elements in **ψ** all can be regarded as the continuous functions of *θ* and *ϕ*. In accordance to ref.^27^, ${\bf{d}}({t}_{1},{t}_{2},{t}_{3}){\boldsymbol{\psi }}$ is the atom of the signal model in Eq. (5). The infinite atomic set is expressed as

6 $${\rm{A}}=\{{\bf{d}}({t}_{1},{t}_{2},{t}_{3}){\boldsymbol{\psi }}|\begin{array}{c}{t}_{1}\equiv \,\sin \,\theta \,\cos \,\varphi {\rm{\Delta }}x/\lambda ,{t}_{2}\equiv \,\sin \,\theta \,\sin \,\varphi {\rm{\Delta }}y/\lambda ,{t}_{3}\equiv \,\cos \,\theta {\rm{\Delta }}z/\lambda ,\\ \theta \in [{0}^{^\circ },{180}^{^\circ }],\varphi \in [{0}^{^\circ },{360}^{^\circ }],{\boldsymbol{\psi }}\in {{\rm{C}}}^{1\times L},{\Vert {\boldsymbol{\psi }}\Vert }_{2}=1\end{array}\}.$$

The atomic ${\ell }_{0}$ norm and the atomic norm of **P** are respectively defined as

7 $${\Vert {\bf{P}}\Vert }_{{\rm{A}},0}=\mathop{{\rm{\inf }}}\limits_{\begin{array}{c}{\bf{d}}({t}_{1i},{t}_{2i},{t}_{3i}){{\boldsymbol{\psi }}}_{i}\in {\rm{A}}\\ {s}_{i}\in {{\rm{R}}}^{+}\end{array}}\{{\rm{I}}|{\bf{P}}=\mathop{\sum }\limits_{i=1}^{{\rm{I}}}{s}_{i}{\bf{d}}({t}_{1i},{t}_{2i},{t}_{3i}){{\boldsymbol{\psi }}}_{i}\}.$$

and

8 $${\Vert {\bf{P}}\Vert }_{{\rm{A}}}=\mathop{{\rm{\inf }}}\limits_{\begin{array}{c}{\bf{d}}({t}_{1i},{t}_{2i},{t}_{3i}){{\boldsymbol{\psi }}}_{i}\in {\rm{A}}\\ {s}_{i}\in {{\rm{R}}}^{+}\end{array}}\{\sum _{i}{s}_{i}|{\bf{P}}=\sum _{i}{s}_{i}{\bf{d}}({t}_{1i},{t}_{2i},{t}_{3i}){{\boldsymbol{\psi }}}_{i}\},$$

where inf denotes the infimum. ${\Vert {\bf{P}}\Vert }_{{\rm{A}}}$ is a convex relaxation of ${\Vert {\bf{P}}\Vert }_{{\rm{A}},0}$^11,18,20^.

Exploiting the source sparsity to solve Eq. (4) can denoise **P**^⭑^ and thus obtain **P**. ${\Vert {\bf{P}}\Vert }_{{\rm{A}},0}$ is the direct metric of source sparsity^11,18,20^. However, it is non-deterministic polynomial-time hard to solve Eq. (4) with the minimization of ${\Vert {\bf{P}}\Vert }_{{\rm{A}},0}$ as a constraint. Replacing ${\Vert {\bf{P}}\Vert }_{{\rm{A}},0}$ with ${\Vert {\bf{P}}\Vert }_{{\rm{A}}}$, we write the reconstruction problem of **P** as

9 $$\hat{{\bf{P}}}=\mathop{{\rm{\arg }}\,{\rm{\min }}}\limits_{{\bf{P}}\in {{\rm{C}}}^{ABC\times L}}{\Vert {\bf{P}}\Vert }_{{\rm{A}}}\,\,{\rm{subject}}\,{\rm{to}}{\Vert {{\bf{P}}}^{\bigstar}-{\bf{P}}\Vert }_{{\rm{F}}}\le \varepsilon ,$$

where *ε* is the estimated noise level. Theoretically, let $\varepsilon ={\Vert {\bf{N}}\Vert }_{{\rm{F}}}$. Equation (9) is a convex optimization problem^26^. For sparse cuboid microphone arrays, ${{\bf{P}}}^{\bigstar}-{\bf{P}}$ in Eq. (9) becomes ${{\bf{P}}}_{{\rm{\Omega }}}^{\bigstar}-{{\bf{P}}}_{{\rm{\Omega }}}$. In this case, the signals induced by sources at full microphones, **P**, are reconstructed from the measurements of partial microphones, ${{\bf{P}}}_{{\rm{\Omega }}}^{\bigstar}$.

### Positive semidefinite programming to solve ANM

For a given column vector ${\bf{u}}=[{u}_{{\alpha }_{1},{\alpha }_{2},{\alpha }_{3}}|({\alpha }_{1},{\alpha }_{2},{\alpha }_{3})\in {\rm{H}}]\in {{\rm{C}}}^{{N}_{u}}$ with ${\rm{H}}=(\{0\}\times \{0\}\times \{0,1,\ldots ,C-1\})$ $\cup$ $(((\{0\}\times \{1,2,\cdots ,B-1\})$ $\cup$ $(\{1,2,\ldots ,A-1\}$ × $\{1-B,2-B,\ldots ,B-1\}))$ × $\{1-C,2-C,\ldots ,C-1\})$ being the halfspace^28^ of $(A-1,B-1,C-1)$ and ${N}_{u}=C+(B-1+(A-1)(2B-1))(2C-1)$, we define a three-fold Toeplitz operator *T*_*bb*_(·) to map **u** into a Hermitian *A* × *A* block Toeplitz matrix:

10 $${T}_{bb}({\bf{u}})=[\begin{array}{cccc}{{\bf{T}}}_{0} & {{\bf{T}}}_{1}^{{\rm{H}}} & \cdots  & {{\bf{T}}}_{A-1}^{{\rm{H}}}\\ {{\bf{T}}}_{1} & {{\bf{T}}}_{0} & \cdots  & {{\bf{T}}}_{A-2}^{{\rm{H}}}\\ \vdots  & \vdots  & \ddots  & \vdots \\ {{\bf{T}}}_{A-1} & {{\bf{T}}}_{A-2} & \cdots  & {{\bf{T}}}_{0}\end{array}],$$

where (·)^H^ denotes the Hermitian operator, each block ${{\bf{T}}}_{{\alpha }_{1}}(0\le {\alpha }_{1}\le A-1)$ is a *B* × *B* block Toeplitz matrix:

11 $${{\bf{T}}}_{{\alpha }_{1}}=[\begin{array}{cccc}{{\bf{T}}}_{{\alpha }_{1},0} & {{\bf{T}}}_{{\alpha }_{1},-1} & \cdots  & {{\bf{T}}}_{{\alpha }_{1},1-B}\\ {{\bf{T}}}_{{\alpha }_{1},1} & {{\bf{T}}}_{{\alpha }_{1},0} & \cdots  & {{\bf{T}}}_{{\alpha }_{1},2-B}\\ \vdots  & \vdots  & \ddots  & \vdots \\ {{\bf{T}}}_{{\alpha }_{1},B-1} & {{\bf{T}}}_{{\alpha }_{1},B-2} & \cdots  & {{\bf{T}}}_{{\alpha }_{1},0}\end{array}],$$

and **T**_0_ is Hermitian, i.e., ${{\bf{T}}}_{0,-{\alpha }_{2}}={{\bf{T}}}_{0,{\alpha }_{2}}^{{\rm{H}}}(0\le {\alpha }_{2}\le B-1)$. Each block ${{\bf{T}}}_{{\alpha }_{1},{\alpha }_{2}}(1-B\le {\alpha }_{2}\le B-1)$ in Eq. (11) is a *C* × *C* Toeplitz matrix:

12 $${{\bf{T}}}_{{\alpha }_{1},{\alpha }_{2}}=[\begin{array}{cccc}{u}_{{\alpha }_{1},{\alpha }_{2},0} & {u}_{{\alpha }_{1},{\alpha }_{2},-1} & \cdots  & {u}_{{\alpha }_{1},{\alpha }_{2},1-C}\\ {u}_{{\alpha }_{1},{\alpha }_{2},1} & {u}_{{\alpha }_{1},{\alpha }_{2},0} & \cdots  & {u}_{{\alpha }_{1},{\alpha }_{2},2-C}\\ \vdots  & \vdots  & \ddots  & \vdots \\ {u}_{{\alpha }_{1},{\alpha }_{2},C-1} & {u}_{{\alpha }_{1},{\alpha }_{2},C-2} & \cdots  & {u}_{{\alpha }_{1},{\alpha }_{2},0}\end{array}],$$

and **T**_0,0_ is Hermitian, i.e., ${u}_{0,0,-{\alpha }_{3}}={u}_{0,0,{\alpha }_{3}}^{\ast }(0\le {\alpha }_{3}\le C-1)$.

**Table 1:** Microphone signal reconstruction error, DOA estimation error and source strength quantification error corresponding to Fig. 1e–h, and consuming time of IPM based SDPT3 solver and ADMM based algorithm.

Microphone array	Approach to solve ANM	$\tfrac{\parallel \hat{{\bf{P}}}-{\bf{P}}{\parallel }_{{\bf{F}}}}{\parallel {\bf{P}}{\parallel }_{{\bf{F}}}}$	$\tfrac{\parallel [\hat{{\boldsymbol{\theta }}},\hat{{\boldsymbol{\varphi }}}]-[{\boldsymbol{\theta }},{\boldsymbol{\varphi }}]{\parallel }_{{\bf{F}}}}{{\bf{2}}{\boldsymbol{I}}}$	$\tfrac{\parallel {\hat{{\bf{s}}}}_{{\boldsymbol{rms}}}-{{\bf{s}}}_{{\boldsymbol{rms}}}{\parallel }_{{\bf{2}}}}{\parallel {{\bf{s}}}_{{\boldsymbol{rms}}}{\parallel }_{{\bf{2}}}}$	Consuming time/s
Standard uniform cuboid array with 343 microphones	IPM based SDPT3 solver (Fig. 1e)	2.45%	0.06%	1.95%	1864
	ADMM based algorithm (Fig. 1g)	3.70%	0.06%	3.36%	169
Sparse cuboid array with 170 microphones	IPM based SDPT3 solver (Fig. 1f)	3.32%	0.04%	2.76%	1863
	ADMM based algorithm (Fig. 1h)	7.17%	0.05%	6.84%	270

We propose the following proposition.

*Proposition:* Denote

13 $$\{\hat{{\bf{u}}},\hat{{\boldsymbol{{\rm E}}}}\}=\mathop{{\rm{\arg }}\,{\rm{\min }}}\limits_{{\bf{u}}\in {{\rm{C}}}^{{N}_{u}},{\boldsymbol{{\rm E}}}\in {{\rm{C}}}^{L\times L}}\frac{1}{2\sqrt{ABC}}({\rm{tr}}({T}_{bb}({\bf{u}}))+{\rm{tr}}({\boldsymbol{{\rm E}}}))\,{\rm{subject}}\,{\rm{to}}\,[\begin{array}{cc}{T}_{bb}({\bf{u}}) & {\bf{P}}\\ {{\bf{P}}}^{{\rm{H}}} & {\boldsymbol{{\rm E}}}\end{array}]\ge 0.$$

and

14 $${\Vert {\bf{P}}\Vert }_{{\rm{T}}}=\frac{1}{2\sqrt{ABC}}({\rm{tr}}({T}_{bb}(\hat{{\bf{u}}}))+{\rm{tr}}(\hat{{\bf{E}}})),$$

where tr(·) represents the trace and ≥0 means the matrix is positive semidefinite. If ${T}_{bb}(\hat{{\bf{u}}})$ admits a Vandermonde decomposition^13,14^, i.e.,

15 $${T}_{bb}(\hat{{\bf{u}}})={\bf{V}}{\boldsymbol{\Sigma }}{{\bf{V}}}^{{\rm{H}}},$$

where ${\bf{V}}=[{\bf{d}}({t}_{11},{t}_{21},{t}_{31}),{\bf{d}}({t}_{12},{t}_{22},{t}_{32}),\ldots ,{\bf{d}}({t}_{1r},{t}_{2r},{t}_{3r})]$, ${\boldsymbol{\Sigma }}={\rm{diag}}([{\sigma }_{{\rm{1}}},{\sigma }_{2},\ldots ,{\sigma }_{r}])$, diag(·) forms a diagonal matrix with diagonal being the vector in the brackets, ${\sigma }_{i}(i=1,2,\ldots ,r)\in {{\rm{R}}}^{+}$, and *r* is the rank of ${T}_{bb}(\hat{{\bf{u}}})$, then $\parallel {\bf{P}}{\parallel }_{{\rm{T}}}=\parallel {\bf{P}}{\parallel }_{{\rm{A}}}$.

The proof about the proposition can be found in Supplementary Note 3. Based on the proposition, the following positive semidefinite programming can be used to characterize the ANM in Eq. (9):

16 $$\begin{array}{rcl}\{\hat{{\bf{u}}},\hat{{\bf{P}}},\hat{{\bf{E}}}\} & = & \mathop{{\rm{\arg }}\,{\rm{\min }}}\limits_{{\bf{u}}\in {{\rm{C}}}^{{N}_{u}},{\bf{P}}\in {{\rm{C}}}^{ABC\times L},{\bf{E}}\in {{\rm{C}}}^{L\times L}}\frac{1}{2\sqrt{ABC}}\times \,({\rm{tr}}({T}_{bb}({\bf{u}}))+{\rm{tr}}({\bf{E}}))\\  &  & {\rm{subject}}\,{\rm{to}}\,[\begin{array}{cc}{T}_{bb}({\bf{u}}) & {\bf{P}}\\ {{\bf{P}}}^{{\rm{H}}} & {\bf{E}}\end{array}]\ge 0,\,\,\parallel {{\bf{P}}}^{\bigstar}-{\bf{P}}{\parallel }_{{\rm{F}}}\le \varepsilon \end{array}$$

The Vandermonde decomposition of ${T}_{bb}(\hat{{\bf{u}}})$ is the precondition to make Eqs (16) and (9) strictly equivalent. References^13,14^ has proved that $r\le \,\min \{A,B,C\}$ is the sufficient condition for ${T}_{bb}(\hat{{\bf{u}}})$ to admit a Vandermonde decomposition. For sparse cuboid microphone arrays, ${{\bf{P}}}^{\bigstar}-{\bf{P}}$ in Eq. (16) becomes ${{\bf{P}}}_{{\rm{\Omega }}}^{\bigstar}-{{\bf{P}}}_{{\rm{\Omega }}}$.

### ADMM to solve positive semidefinite programming

Because ${{\bf{P}}}^{\bigstar}-{\bf{P}}$ is a special case of ${{\bf{P}}}_{{\rm{\Omega }}}^{\bigstar}-{{\bf{P}}}_{{\rm{\Omega }}}$ with Ω including the indices of the full microphones, we conduct the derivation on ${{\bf{P}}}_{{\rm{\Omega }}}^{\bigstar}-{{\bf{P}}}_{{\rm{\Omega }}}$. We reformulate Eq. (16) as

17 $$\begin{array}{rcl}\{\hat{{\bf{u}}},\hat{{\bf{P}}},\hat{{\bf{E}}},\hat{{\bf{Z}}}\} & = & \mathop{{\rm{\arg }}\,{\rm{\min }}}\limits_{\begin{array}{c}{\bf{u}}\in {{\rm{C}}}^{{N}_{u}},{\bf{P}}\in {{\rm{C}}}^{ABC\times L},{\bf{E}}\in {{\rm{C}}}^{L\times L}\\ {\bf{Z}}\in {{\rm{C}}}^{(ABC+L)\times (ABC+L)}\end{array}}\frac{{\rm{1}}}{{\rm{2}}}{\Vert {{\bf{P}}}_{{\rm{\Omega }}}^{\bigstar}-{{\bf{P}}}_{{\rm{\Omega }}}\Vert }_{{\rm{F}}}^{{\rm{2}}}+\,\frac{\tau }{2\sqrt{ABC}}({\rm{tr}}({T}_{bb}({\bf{u}}))+{\rm{tr}}({\bf{E}}))\\  &  & {\rm{subject}}\,{\rm{to}}\,{\bf{Z}}=[\begin{array}{cc}{T}_{bb}({\bf{u}}) & {\bf{P}}\\ {{\bf{P}}}^{{\rm{H}}} & {\bf{E}}\end{array}],{\bf{Z}}\ge 0\end{array}$$

where *τ* is a regularization parameter and **Z** is an auxiliary matrix. The augmented Lagrangian function of Eq. (17) is

18 $$\begin{array}{rcl}{{\rm{L}}}_{\rho }({\bf{E}},{\bf{u}},{\bf{P}},{\bf{Z}},{\boldsymbol{\Lambda }}) & = & \frac{{\rm{1}}}{{\rm{2}}}{\Vert {{\bf{P}}}_{{\rm{\Omega }}}^{\bigstar}-{{\bf{P}}}_{{\rm{\Omega }}}\Vert }_{{\rm{F}}}^{{\rm{2}}}+\frac{\tau }{2\sqrt{ABC}}({\rm{tr}}({T}_{bb}({\bf{u}}))+{\rm{tr}}({\bf{E}}))\\  &  & +\langle {\boldsymbol{\Lambda }},{\bf{Z}}-[\begin{array}{cc}{T}_{bb}({\bf{u}}) & {\bf{P}}\\ {{\bf{P}}}^{{\rm{H}}} & {\bf{E}}\end{array}]\rangle +\frac{\rho }{2}{\Vert {\bf{Z}}-[\begin{array}{cc}{T}_{bb}({\bf{u}}) & {\bf{P}}\\ {{\bf{P}}}^{{\rm{H}}} & {\bf{E}}\end{array}]\Vert }_{{\rm{F}}}^{{\rm{2}}}\end{array},$$

where the Hermitian matrix ${\boldsymbol{\Lambda }}\in {{\rm{C}}}^{(ABC+L)\times (ABC+L)}$ is the Lagrangian multiplier, $\rho  > 0$ is the penalty parameter and 〈·,·〉 denotes the inner product. The ADMM solves Eq. (17) iteratively. Initializing ${{\bf{Z}}}^{{\rm{0}}}={{\boldsymbol{\Lambda }}}^{0}={\bf{0}}$, the updates in $(q+{\rm{1}}){\rm{th}}$ iteration are as follows:

19 $$\{{{\bf{E}}}^{q+1},{{\bf{u}}}^{q+1},{{\bf{P}}}^{q+1}\}=\mathop{{\rm{\arg }}\,{\rm{\min }}}\limits_{{\bf{E}}\in {{\rm{C}}}^{L\times L},{\bf{u}}\in {{\rm{C}}}^{{N}_{u}},{\bf{P}}\in {{\rm{C}}}^{ABC\times L}}{{\rm{L}}}_{\rho }({\bf{E}},{\bf{u}},{\bf{P}},{{\bf{Z}}}^{q},{{\boldsymbol{\Lambda }}}^{q}),$$

20 $${{\bf{Z}}}^{q+{\rm{1}}}=\mathop{{\rm{\arg }}\,{\rm{\min }}}\limits_{{\bf{Z}}\ge 0}{{\rm{L}}}_{\rho }({{\bf{E}}}^{q+1},{{\bf{u}}}^{q+1},{{\bf{P}}}^{q+1},{\bf{Z}},{{\boldsymbol{\Lambda }}}^{q}),$$

21 $${{\boldsymbol{\Lambda }}}^{q+1}={{\boldsymbol{\Lambda }}}^{q}+\rho ({{\bf{Z}}}^{q+1}-[\begin{array}{cc}{T}_{bb}({{\bf{u}}}^{q+1}) & {{\bf{P}}}^{q+1}\\ {({{\bf{P}}}^{q+1})}^{{\rm{H}}} & {{\bf{E}}}^{q+1}\end{array}]).$$

Introduce the partitions

22 $${{\boldsymbol{\Lambda }}}^{q}=[\begin{array}{cc}{{\boldsymbol{\Lambda }}}_{0}^{q}\in {{\rm{C}}}^{ABC\times ABC} & {{\boldsymbol{\Lambda }}}_{1}^{q}\in {{\rm{C}}}^{ABC\times L}\\ {{\boldsymbol{\Lambda }}}_{1}^{q{\rm{H}}}\in {{\rm{C}}}^{L\times ABC} & {{\boldsymbol{\Lambda }}}_{2}^{q}\in {{\rm{C}}}^{L\times L}\end{array}]\,{\rm{and}}\,{{\bf{Z}}}^{q}=[\begin{array}{cc}{{\bf{Z}}}_{0}^{q}\in {{\rm{C}}}^{ABC\times ABC} & {{\bf{Z}}}_{1}^{q}\in {{\rm{C}}}^{ABC\times L}\\ {{\bf{Z}}}_{1}^{q{\rm{H}}}\in {{\rm{C}}}^{L\times ABC} & {{\bf{Z}}}_{2}^{q}\in {{\rm{C}}}^{L\times L}\end{array}]$$

Denote by ${{\boldsymbol{\Lambda }}}_{{\rm{1}}{\rm{\Omega }}}^{q}\in {{\rm{C}}}^{|{\rm{\Omega }}|\times L}$ and ${{\bf{Z}}}_{1{\rm{\Omega }}}^{q}\in {{\rm{C}}}^{|{\rm{\Omega }}|\times L}$, respectively, the matrices of the rows in ${{\boldsymbol{\Lambda }}}_{{\rm{1}}}^{q}$ and ${{\bf{Z}}}_{1}^{q}$ corresponding to the retained microphones, ${{\rm{\Omega }}}^{c}$ the set of the indices of the unretained microphones, $|{{\rm{\Omega }}}^{c}|$ the cardinality of ${{\rm{\Omega }}}^{c}$, ${{\bf{P}}}_{{{\rm{\Omega }}}^{c}}\in {{\rm{C}}}^{|{{\rm{\Omega }}}^{c}|\times L}$ the matrix of the signals induced by sources at the unretained microphones, ${{\boldsymbol{\Lambda }}}_{{1{\rm{\Omega }}}^{c}}^{q}\in {{\rm{C}}}^{|{{\rm{\Omega }}}^{c}|\times L}$ and ${{\bf{Z}}}_{{1{\rm{\Omega }}}^{c}}^{q}\in {{\rm{C}}}^{|{{\rm{\Omega }}}^{c}|\times L}$, respectively, the matrices of the rows in ${{\boldsymbol{\Lambda }}}_{{\rm{1}}}^{q}$ and ${{\bf{Z}}}_{1}^{q}$ corresponding to the unretained microphones, and ${{\bf{I}}}_{1}\in {{\rm{R}}}^{L\times L}$ and ${{\bf{I}}}_{2}\in {{\rm{R}}}^{ABC\times ABC}$, both, the identity matrices. It can be derived that the variable updates in Eq. (19) have the following closed forms:

23 $${{\bf{E}}}^{q+1}={{\bf{Z}}}_{2}^{q}+\frac{1}{\rho }({{\boldsymbol{\Lambda }}}_{2}^{q}-\frac{\tau }{2\sqrt{ABC}}{{\bf{I}}}_{1}),$$

24 $${T}_{bb}{({\bf{u}})}^{q+1}={{\bf{Z}}}_{0}^{q}+\frac{1}{\rho }({{\boldsymbol{\Lambda }}}_{0}^{q}-\frac{\tau }{2\sqrt{ABC}}{{\bf{I}}}_{2}),$$

25 $${{\bf{P}}}_{{\rm{\Omega }}}^{q+1}=\frac{{\rm{1}}}{1+2\rho }({{\bf{P}}}_{{\rm{\Omega }}}^{\bigstar}+{\rm{2}}{{\boldsymbol{\Lambda }}}_{1{\rm{\Omega }}}^{q}+{\rm{2}}\rho {{\bf{Z}}}_{1{\rm{\Omega }}}^{q}),$$

26 $${{\bf{P}}}_{{{\rm{\Omega }}}^{c}}^{q+1}={{\bf{Z}}}_{{1{\rm{\Omega }}}^{c}}^{q}+\frac{1}{\rho }{{\boldsymbol{\Lambda }}}_{{1{\rm{\Omega }}}^{c}}^{q}.$$

The derivation can be found in Supplementary Note 4. Let $${\bf{M}}=\mathrm{diag}([ABC,AB(C-1),\cdots ,AB,[A(B-1)$$, $$A(B-2),\cdots ,A,[A-1,A-2,\cdots ,1]\otimes [1,2,\cdots ,B,B-1,\cdots ,1]]$$ $$\otimes [1,2,\cdots ,C,C-1,\cdots ,1]])$$ $$\in {{\rm{R}}}^{{N}_{u}\times {N}_{u}}$$ and $${T}_{bb}^{\ast }$$(·) be the adjoint of *T*_*bb*_(·). For a given matrix $${\bf{A}}\in {{\rm{C}}}^{ABC\times ABC}$$, $${T}_{bb}^{\ast }({\bf{A}})=$$[$${\rm{tr}}(({{\rm{\Theta }}}_{{\alpha }_{1}}\otimes {{\rm{\Theta }}}_{{\alpha }_{2}}\otimes {{\rm{\Theta }}}_{{\alpha }_{3}}){\bf{A}})$$$$|({\alpha }_{1},{\alpha }_{2},{\alpha }_{3})\in {\rm{H}}$$]$$\in {{\rm{C}}}^{{N}_{u}}$$, where $${{\rm{\Theta }}}_{\alpha }$$ is an elementary Toeplitz matrix with ones on the *α*th diagonal and zeros elsewhere. Then,

27 $${{\bf{u}}}^{q+1}={{\bf{M}}}^{-1}{T}_{bb}^{\ast }({T}_{bb}{({\bf{u}})}^{q+1}).$$

We rewritten the update of **Z** in Eq. (20) as

28 $${{\bf{Z}}}^{q+{\rm{1}}}=\mathop{{\rm{\arg }}\,{\rm{\min }}}\limits_{{\bf{Z}}\ge 0}{\Vert {\bf{Z}}-[\begin{array}{cc}{T}_{bb}({{\bf{u}}}^{q+1}) & {{\bf{P}}}^{q+1}\\ {({{\bf{P}}}^{q+1})}^{{\rm{H}}} & {{\bf{E}}}^{q+1}\end{array}]+\frac{{{\boldsymbol{\Lambda }}}^{q}}{\rho }\Vert }_{{\rm{F}}}^{2},$$

which can be performed by conducting the eigenvalue decomposition of the Hermitian matrix $$[\begin{array}{cc}{T}_{bb}({{\bf{u}}}^{q+1}) & {{\bf{P}}}^{q+1}\\ {({{\bf{P}}}^{q+1})}^{{\rm{H}}} & {{\bf{E}}}^{q+1}\end{array}]-\frac{{{\boldsymbol{\Lambda }}}^{q}}{\rho }$$ and setting all negative eigenvalues to zero.

A reasonable termination criterion of ADMM is that the primal and dual residuals or infeasibilities must be small^22–24^. For the semidefinite programming, the primal and dual infeasibilities are recommended for use^23,24^. According to refs^23,24^, the primal and dual infeasibilities after *q* iterations for the current problem can be respectively defined as

29 $${{\rm{pri}}}_{-}{{\rm{\inf }}}^{q}=\frac{{\Vert {{\bf{Z}}}^{q}-[\begin{array}{cc}{T}_{bb}({{\bf{u}}}^{q}) & {{\bf{P}}}^{q}\\ {({{\bf{P}}}^{q})}^{{\rm{H}}} & {{\bf{E}}}^{q}\end{array}]\Vert }_{{\rm{F}}}}{ABC+L+\,{\rm{\max }}({\Vert {{\bf{Z}}}^{q}\Vert }_{{\rm{F}}},{\Vert [\begin{array}{cc}{T}_{bb}({{\bf{u}}}^{q}) & {{\bf{P}}}^{q}\\ {({{\bf{P}}}^{q})}^{{\rm{H}}} & {{\bf{E}}}^{q}\end{array}]\Vert }_{{\rm{F}}})}.$$

and

30 $${{\rm{dual}}}_{-}{{\rm{\inf }}}^{q}=\frac{\rho \sqrt{{\Vert {T}_{bb}^{\ast }({{\bf{Z}}}_{0}^{q}-{{\bf{Z}}}_{0}^{q-1})\Vert }_{{\rm{2}}}^{{\rm{2}}}+{\Vert {{\bf{Z}}}_{{\rm{1}}}^{q}-{{\bf{Z}}}_{{\rm{1}}}^{q-1}\Vert }_{{\rm{F}}}^{{\rm{2}}}+{\Vert {{\bf{Z}}}_{2}^{q}-{{\bf{Z}}}_{2}^{q-1}\Vert }_{{\rm{F}}}^{{\rm{2}}}}}{\sqrt{{N}_{u}+ABCL+{L}^{2}}+\rho \sqrt{{\Vert {T}_{bb}^{\ast }({{\boldsymbol{\Lambda }}}_{0}^{q}-{{\boldsymbol{\Lambda }}}_{0}^{q-1})\Vert }_{{\rm{2}}}^{{\rm{2}}}+{\Vert {{\boldsymbol{\Lambda }}}_{{\rm{1}}}^{q}-{{\boldsymbol{\Lambda }}}_{{\rm{1}}}^{q-1}\Vert }_{{\rm{F}}}^{{\rm{2}}}+{\Vert {{\boldsymbol{\Lambda }}}_{2}^{q}-{{\boldsymbol{\Lambda }}}_{2}^{q-1}\Vert }_{{\rm{F}}}^{{\rm{2}}}}}.$$

The algorithm terminates when $\max \,\{{{\rm{pri}}}_{-}{{\rm{\inf }}}^{q},{{\rm{dual}}}_{-}{{\rm{\inf }}}^{q}\}$ is less than a pre-set tolerance level or the pre-set maximum number of iterations is reached. Another reasonable termination criterion is that the relative change of the result at two consecutive iterations is small enough, which means the result has converged.

### DOA estimation via MaPP

${T}_{bb}(\hat{{\bf{u}}})$ contains the DOA information of sources. That can be retrieved via the MaPP method^14,20^. The concrete steps are:

1) Conduct the eigendecomposition on ${T}_{bb}(\hat{{\bf{u}}})$:

31 $${T}_{bb}(\hat{{\bf{u}}})={{\bf{U}}}_{{\rm{1}}}{{\bf{C}}}_{{\rm{1}}}{{\bf{U}}}_{{\rm{1}}}^{{\rm{H}}},$$

where ${{\bf{U}}}_{{\rm{1}}}\in {{\rm{C}}}^{ABC\times ABC}$ is the unitary matrix whose columns are the eigenvectors of ${T}_{bb}(\hat{{\bf{u}}})$ and ${{\bf{C}}}_{{\rm{1}}}\in {{\rm{R}}}^{ABC\times ABC}$ is the diagonal matrix whose diagonal elements are the corresponding eigenvalues. Estimate the total number of sources as the number of eigenvalues larger than a given threshold and denote it by $\hat{I}$. Denote by ${{\bf{C}}}_{{\rm{1}}e}\in {{\rm{R}}}^{\hat{I}\times \hat{I}}$ the diagonal matrix with diagonal elements being the square roots of the $\hat{I}$ larger eigenvalues and ${{\bf{U}}}_{{\rm{1}}e}\in {{\rm{C}}}^{ABC\times \hat{I}}$ the matrix with columns being the corresponding eigenvectors. Let ${{\bf{Y}}}_{{\rm{1}}}={{\bf{U}}}_{{\rm{1}}e}{{\bf{C}}}_{{\rm{1}}e}\in {{\rm{C}}}^{ABC\times \hat{I}}$.

2) Delete the last *BC* rows of ${{\bf{Y}}}_{{\rm{1}}}$ to obtain ${{\bf{Y}}}_{{\rm{1}}u}\in {{\rm{C}}}^{(A-1)BC\times \hat{I}}$ and the first *BC* rows to obtain ${{\bf{Y}}}_{{\rm{1}}d}\in {{\rm{C}}}^{(A-1)BC\times \hat{I}}$. Compute the generalized eigenvalues of the matrix pencil $({{\bf{Y}}}_{{\rm{1}}d},{{\bf{Y}}}_{{\rm{1}}u})$ to obtain $\{{e}^{j2\pi {t}_{1m}}|m=1,2,\cdots ,\hat{I}\}$.

3) Let ${\boldsymbol{\rho }}(\alpha )\in {{\rm{R}}}^{ABC}$ be the column vector with one on the *α*th position and zeros elsewhere, ${{\boldsymbol{\rho }}}_{{\alpha }_{1},{\alpha }_{2},{\alpha }_{3}}={\boldsymbol{\rho }}({\alpha }_{1}C+{\alpha }_{2}AC+{\alpha }_{3}+1)$, ${{\bf{P}}}_{{\alpha }_{1},{\alpha }_{2}}=[{{\boldsymbol{\rho }}}_{{\alpha }_{1},{\alpha }_{2},0},{{\boldsymbol{\rho }}}_{{\alpha }_{1},{\alpha }_{2},1},\cdots ,{{\boldsymbol{\rho }}}_{{\alpha }_{1},{\alpha }_{2},C-1}]\in {{\rm{R}}}^{ABC\times C}$, ${{\bf{P}}}_{{\alpha }_{1}}=[{{\bf{P}}}_{{\alpha }_{1},0},{{\bf{P}}}_{{\alpha }_{1},1},\cdots ,{{\bf{P}}}_{{\alpha }_{1},B-1}]\in {{\rm{R}}}^{ABC\times BC}$, and ${\bf{P}}=[{{\bf{P}}}_{0},{{\bf{P}}}_{1},\cdots ,{{\bf{P}}}_{A-1}]\in {{\rm{R}}}^{ABC\times ABC}$. ${\bf{P}}{T}_{bb}(\hat{{\bf{u}}}){{\bf{P}}}^{{\rm{T}}}$ permutes the elements of ${T}_{bb}(\hat{{\bf{u}}})$ to obtain a new three-fold Toeplitz matrix. The first fold contains *B* × *B* big blocks. The second fold contains *A* × *A* small blocks. The third fold is the *C* × *C* matrix. Conduct the eigendecomposition on ${\bf{P}}{T}_{bb}(\hat{{\bf{u}}}){{\bf{P}}}^{{\rm{T}}}$:

32 $${\bf{P}}{T}_{bb}(\hat{{\bf{u}}}){{\bf{P}}}^{{\rm{T}}}={{\bf{U}}}_{2}{{\bf{C}}}_{2}{{\bf{U}}}_{2}^{{\rm{H}}},$$

where ${{\bf{U}}}_{2}\in {{\rm{C}}}^{ABC\times ABC}$ is the unitary matrix whose columns are the eigenvectors of ${\bf{P}}{T}_{bb}(\hat{{\bf{u}}}){{\bf{P}}}^{{\rm{T}}}$ and ${{\bf{C}}}_{{\rm{2}}}\in {{\rm{R}}}^{ABC\times ABC}$ is the diagonal matrix whose diagonal elements are the corresponding eigenvalues. Denote by ${{\bf{C}}}_{{\rm{2}}e}\in {{\rm{R}}}^{\hat{I}\times \hat{I}}$ the diagonal matrix with diagonal elements being the square roots of the $\hat{I}$ larger eigenvalues and ${{\bf{U}}}_{{\rm{2}}e}\in {{\rm{C}}}^{ABC\times \hat{I}}$ the matrix with columns being the corresponding eigenvectors. Let ${{\bf{Y}}}_{{\rm{2}}}={{\bf{U}}}_{{\rm{2}}e}{{\bf{C}}}_{{\rm{2}}e}\in {{\rm{C}}}^{ABC\times \hat{I}}$.

4) Delete the last *AC* rows of ${{\bf{Y}}}_{{\rm{2}}}$ to obtain ${{\bf{Y}}}_{2u}\in {{\rm{C}}}^{A(B-1)C\times \hat{I}}$ and the first *AC* rows to obtain ${{\bf{Y}}}_{2d}\in {{\rm{C}}}^{A(B-1)C\times \hat{I}}$. Compute the generalized eigenvalues of the matrix pencil $({{\bf{Y}}}_{{\rm{2}}d},{{\bf{Y}}}_{{\rm{2}}u})$ to obtain $\{{e}^{j2\pi {t}_{2n}}|n=1,2,\cdots ,\hat{I}\}$.

5) Let ${\tilde{{\boldsymbol{\rho }}}}_{{\alpha }_{1},{\alpha }_{2},{\alpha }_{3}}={\boldsymbol{\rho }}(1+{\alpha }_{1}+{\alpha }_{2}A+{\alpha }_{3}AB)$, ${\tilde{{\bf{P}}}}_{{\alpha }_{1},{\alpha }_{2}}=[{\tilde{{\boldsymbol{\rho }}}}_{{\alpha }_{1},{\alpha }_{2},0},{\tilde{{\boldsymbol{\rho }}}}_{{\alpha }_{1},{\alpha }_{2},1},\cdots ,{\tilde{{\boldsymbol{\rho }}}}_{{\alpha }_{1},{\alpha }_{2},C-1}]\in {{\rm{R}}}^{ABC\times C}$, ${\tilde{{\bf{P}}}}_{{\alpha }_{1}}=[{\tilde{{\bf{P}}}}_{{\alpha }_{1},0},{\tilde{{\bf{P}}}}_{{\alpha }_{1},1},\cdots ,{\tilde{{\bf{P}}}}_{{\alpha }_{1},B-1}]\in {{\rm{R}}}^{ABC\times BC}$, and $\tilde{{\bf{P}}}=[{\tilde{{\bf{P}}}}_{0},{\tilde{{\bf{P}}}}_{1},\cdots ,{\tilde{{\bf{P}}}}_{A-1}]\in {{\rm{R}}}^{ABC\times ABC}$. $\tilde{{\bf{P}}}{T}_{bb}(\hat{{\bf{u}}}){\tilde{{\bf{P}}}}^{{\rm{T}}}$ permutes the elements of ${T}_{bb}(\hat{{\bf{u}}})$ to obtain another new three-fold Toeplitz matrix. The first fold contains *C* × *C* big blocks. The second fold contains *B* × *B* small blocks. The third fold is the *A* × *A* matrix. Conduct the eigendecomposition on $\tilde{{\bf{P}}}{T}_{bb}(\hat{{\bf{u}}}){\tilde{{\bf{P}}}}^{{\rm{T}}}$:

33 $$\tilde{{\bf{P}}}{T}_{bb}(\hat{{\bf{u}}}){\tilde{{\bf{P}}}}^{{\rm{T}}}={{\bf{U}}}_{3}{{\bf{C}}}_{3}{{\bf{U}}}_{3}^{{\rm{H}}},$$

where ${{\bf{U}}}_{{\rm{3}}}\in {{\rm{C}}}^{ABC\times ABC}$ is the unitary matrix whose columns are the eigenvectors of $\tilde{{\bf{P}}}{T}_{bb}(\hat{{\bf{u}}}){\tilde{{\bf{P}}}}^{{\rm{T}}}$ and ${{\bf{C}}}_{{\rm{3}}}\in {{\rm{R}}}^{ABC\times ABC}$ is the diagonal matrix whose diagonal elements are the corresponding eigenvalues. Denote by ${{\bf{C}}}_{{\rm{3}}e}\in {{\rm{R}}}^{\hat{I}\times \hat{I}}$ the diagonal matrix with diagonal elements being the square roots of the $\hat{I}$ larger eigenvalues and ${{\bf{U}}}_{{\rm{3}}e}\in {{\rm{C}}}^{ABC\times \hat{I}}$ the matrix with columns being the corresponding eigenvectors. Let ${{\bf{Y}}}_{{\rm{3}}}={{\bf{U}}}_{{\rm{3}}e}{{\bf{C}}}_{{\rm{3}}e}\in {{\rm{C}}}^{ABC\times \hat{I}}$.

6) Delete the last *AB* rows of ${{\bf{Y}}}_{3}$ to obtain ${{\bf{Y}}}_{{\rm{3}}u}\in {{\rm{C}}}^{AB(C-1)\times \hat{I}}$ and the first *AB* rows to obtain ${{\bf{Y}}}_{{\rm{3}}d}\in {{\rm{C}}}^{AB(C-1)\times \hat{I}}$. Compute the generalized eigenvalues of the matrix pencil $({{\bf{Y}}}_{{\rm{3}}d},{{\bf{Y}}}_{{\rm{3}}u})$ to obtain $\{{e}^{j2\pi {t}_{{\rm{3}}o}}|o=1,2,\cdots ,\hat{I}\}$.

7) Compute the function

34 $$(f(m),g(m))=\mathop{{\rm{\arg }}\,{\rm{\max }}}\limits_{(n,o)\in \{1,2,\cdots ,\hat{I}\}\times \{1,2,\cdots ,\hat{I}\}}{\Vert {{\bf{U}}}_{{\rm{1}}e}^{{\rm{H}}}{\bf{d}}({t}_{1m},{t}_{2n},{t}_{3o})\Vert }_{2}^{2}$$

to pair *m*, *n* and *o*, and then obtain the pairs $\{({e}^{j2\pi {t}_{1m}},{e}^{j2\pi {t}_{2f(m)}},{e}^{j2\pi {t}_{{\rm{3}}g(m)}})|m=1,2,\cdots ,\hat{I}\}$ (denoted as $\{({e}^{j2\pi {t}_{1i}},{e}^{j2\pi {t}_{2i}},{e}^{j2\pi {t}_{{\rm{3}}i}})|i=1,2,\cdots ,\hat{I}\}$ for simplicity).

8) Compute $\{({t}_{1i},{t}_{2i},{t}_{{\rm{3}}i})|i=1,2,\cdots ,\hat{I}\}$ according to ${t}_{1i}={\rm{Im}}(\mathrm{ln}({e}^{j2\pi {t}_{1i}}))/2\pi$, ${t}_{2i}={\rm{Im}}(\mathrm{ln}({e}^{j2\pi {t}_{2i}}))/2\pi$ and ${t}_{{\rm{3}}i}={\rm{Im}}(\mathrm{ln}({e}^{j2\pi {t}_{{\rm{3}}i}}))/2\pi$, where Im(·) denotes the imaginary part. Compute $\{({\theta }_{i},{\varphi }_{i})|i=1,2,\cdots ,\hat{I}\}$ according to the relationship between $({t}_{1i},{t}_{2i},{t}_{{\rm{3}}i})$ and $({\theta }_{i},{\varphi }_{i})$, i.e., ${t}_{1i}\equiv \,\sin \,{\theta }_{i}\,\cos \,{\varphi }_{i}{\rm{\Delta }}x/\lambda$, ${t}_{2i}\equiv \,\sin \,{\theta }_{i}\,\sin \,{\varphi }_{i}{\rm{\Delta }}y/\lambda$ and ${t}_{3i}\equiv \,\cos \,{\theta }_{i}{\rm{\Delta }}z/\lambda$.

### Source strength quantification

After computing the sensing matrix $$\hat{{\bf{D}}}=$$[$${\bf{d}}({t}_{11},{t}_{21},{t}_{31}),{\bf{d}}({t}_{12},{t}_{22},{t}_{32}),$$$$\cdots ,{\bf{d}}({t}_{1\hat{I}},{t}_{2\hat{I}},{t}_{3\hat{I}})$$]$$\in {{\rm{C}}}^{ABC\times \hat{I}}$$ according to the estimated DOAs, we quantify the matrix $$\hat{{\bf{S}}}={[{{\bf{s}}}_{1}^{{\rm{T}}},{{\bf{s}}}_{2}^{{\rm{T}}},\cdots ,{{\bf{s}}}_{\hat{I}}^{{\rm{T}}}]}^{{\rm{T}}}\in {{\rm{C}}}^{\hat{I}\times L}$$ composed by the strength of each source under each snapshot as

35 $$\hat{{\bf{S}}}={\hat{{\bf{D}}}}^{+}\hat{{\bf{P}}},$$

where ·^+^ denotes the pseudo-inverse.

### IRANM

Establish the metric

36 $${{\rm{M}}}^{\kappa }({\bf{P}})=\mathop{{\rm{\min }}}\limits_{{\bf{u}}\in {{\rm{C}}}^{{N}_{u}},{\bf{E}}\in {{\rm{C}}}^{L\times L}}\frac{1}{2\sqrt{ABC}}(\mathrm{ln}|{T}_{bb}({\bf{u}})+\kappa {{\bf{I}}}_{{\rm{2}}}|+{\rm{tr}}({\bf{E}}))\,{\rm{subject}}\,{\rm{to}}\,[\begin{array}{cc}{T}_{bb}({\bf{u}}) & {\bf{P}}\\ {{\bf{P}}}^{{\rm{H}}} & {\bf{E}}\end{array}]\ge 0,$$

where |·| denotes the determinant of a matrix and $\kappa  > 0$ is a regularization parameter. ${{\rm{M}}}^{\kappa }({\bf{P}})$ has the following properties under a certain condition^11,14,18^: (1) ${{\rm{M}}}^{\kappa }({\bf{P}})$ is on the order of $({\Vert {\bf{P}}\Vert }_{{\rm{A}},0}/2\sqrt{ABC}-\sqrt{ABC}/2)\mathrm{ln}\,{\kappa }^{-{\rm{1}}}$ as *κ* approaches 0 if ${\Vert {\bf{P}}\Vert }_{{\rm{A}},0} < ABC$, i.e., $$\begin{array}{c}\mathrm{lim}\\ \kappa \to 0\end{array}{{\rm{M}}}^{\kappa }({\bf{P}})/(({\Vert {\bf{P}}\Vert }_{{\rm{A}},0}/2\sqrt{ABC}-\sqrt{ABC}/2)\mathrm{ln}\,{\kappa }^{-1})=1$$; (2) ${{\rm{M}}}^{\kappa }({\bf{P}})-(\sqrt{ABC}/2)\mathrm{ln}\,\kappa$ is on the order of ${\Vert {\bf{P}}\Vert }_{{\rm{A}}}{\kappa }^{-1/2}$ as *κ* approaches +∞, i.e., $$\begin{array}{c}{\rm{l}}{\rm{i}}{\rm{m}}\\ \kappa \to +\,{\rm{\infty }}\end{array}({{\rm{M}}}^{\kappa }({\bf{P}})-(\sqrt{ABC}/2){\rm{l}}{\rm{n}}\,\kappa )/({\Vert {\bf{P}}\Vert }_{{\rm{A}}}{\kappa }^{-1/2})=1$$; (3) Denote by ${\hat{{\bf{u}}}}_{\kappa \to 0}$ the optimal variable when *κ* approaches 0. The smallest $ABC-{\Vert {\bf{P}}\Vert }_{{\rm{A}},0}$ eigenvalues of ${T}_{bb}({\hat{{\bf{u}}}}_{\kappa \to 0})$ are either 0 or approach 0, i.e., only ${\Vert {\bf{P}}\Vert }_{{\rm{A}},0}$ eigenvalues are large. According to the first property, minimizing ${{\rm{M}}}^{\kappa }({\bf{P}})$ is equivalent to minimizing ${\Vert {\bf{P}}\Vert }_{{\rm{A}},0}$ as *κ* approaches 0. According to the second property, minimizing ${{\rm{M}}}^{\kappa }({\bf{P}})$ is equivalent to minimizing ${\Vert {\bf{P}}\Vert }_{{\rm{A}}}$ as *κ* approaches +∞. This means ${{\rm{M}}}^{\kappa }({\bf{P}})$ serves as a bridge between ${\Vert {\bf{P}}\Vert }_{{\rm{A}},0}$ and ${\Vert {\bf{P}}\Vert }_{{\rm{A}}}$, and can enhance sparsity compared with ${\Vert {\bf{P}}\Vert }_{{\rm{A}}}$. According to the third property, the sufficient condition for ${T}_{bb}({\hat{{\bf{u}}}}_{\kappa \to 0})$ to admit a Vandermonde decomposition can be guaranteed.

Replacing ${\Vert {\bf{P}}\Vert }_{{\rm{A}}}$ with ${{\rm{M}}}^{\kappa }({\bf{P}})$, we reformulate the reconstruction problem of **P** as

37 $$\hat{{\bf{P}}}=\mathop{{\rm{\arg }}\,{\rm{\min }}}\limits_{{\bf{P}}\in {{\rm{C}}}^{ABC\times L}}{{\rm{M}}}^{\kappa }({\bf{P}})\,\,{\rm{subject}}\,{\rm{to}}{\Vert {{\bf{P}}}^{\bigstar}-{\bf{P}}\Vert }_{{\rm{F}}}\le \varepsilon .$$

Simultaneous Eqs (36) and (37) yield

38 $$\begin{array}{rcl}\{\hat{{\bf{u}}},\hat{{\bf{P}}},\hat{{\bf{E}}}\} & = & \mathop{{\rm{\arg }}\,{\rm{\min }}}\limits_{{\bf{u}}\in {{\rm{C}}}^{{N}_{u}},{\bf{P}}\in {{\rm{C}}}^{ABC\times L},{\bf{E}}\in {{\rm{C}}}^{L\times L}}\frac{1}{2\sqrt{ABC}}\times \,(\mathrm{ln}|{T}_{bb}({\bf{u}})+\kappa {{\bf{I}}}_{2}|+{\rm{tr}}({\bf{E}}))\\  &  & {\rm{subject}}\,{\rm{to}}[\begin{array}{cc}{T}_{bb}({\bf{u}}) & {\bf{P}}\\ {{\bf{P}}}^{{\rm{H}}} & {\bf{E}}\end{array}]\ge 0,{\Vert {{\bf{P}}}^{\bigstar}-{\bf{P}}\Vert }_{{\rm{F}}}\le \varepsilon \end{array}.$$

In Eq. (38), $\mathrm{ln}|{T}_{bb}({\bf{u}})+\kappa {\bf{I}}|$ is a concave function of **u**, while ${\rm{tr}}({\bf{E}})$ is a convex function of **E**. To minimize such a concave + convex function, an effective algorithm is the majorization-minimization^29,30^. The algorithm solves the optimal variable and the optimal value of the objective function iteratively. Two steps are involved in each iteration. In the first majorization step, a surrogate function is constructed to locally approximate the objective function. The surrogate function should be no smaller than the objective function, and the equality holds at the current optimal variable. Then in the minimization step, the surrogate function is minimized to obtain a new optimal variable. Let ${\hat{{\bf{u}}}}^{k}$ be the optimal variable, ${\kappa }^{k}$ be the regularization parameter, ${{\bf{W}}}^{k}\equiv {({T}_{bb}({\hat{{\bf{u}}}}^{k})+{\kappa }^{k}{{\bf{I}}}_{2})}^{-1}\in {{\rm{C}}}^{ABC\times ABC}$ be the weighting matrix, determined by the *k*th iteration. The surrogate function in $(k+{\rm{1}}){\rm{th}}$ iteration can be constructed as

39 $$\frac{1}{2\sqrt{ABC}}(\mathrm{ln}|{T}_{bb}({\hat{{\bf{u}}}}^{k})+{\kappa }^{k}{{\bf{I}}}_{{\rm{2}}}|+{\rm{tr}}({{\bf{W}}}^{k}{T}_{bb}({\bf{u}}-{\hat{{\bf{u}}}}^{k}))+{\rm{tr}}({\bf{E}}))=\frac{1}{2\sqrt{ABC}}({\rm{tr}}({{\bf{W}}}^{k}{T}_{bb}({\bf{u}}))+{\rm{tr}}({\bf{E}}))+{c}^{k},$$

where $\mathrm{ln}|{T}_{bb}({\hat{{\bf{u}}}}^{k})+{\kappa }^{k}{{\bf{I}}}_{{\rm{2}}}|+{\rm{tr}}({{\bf{W}}}^{k}{T}_{bb}({\bf{u}}-{\hat{{\bf{u}}}}^{k}))$ is the tangent plane of $\mathrm{ln}|{T}_{bb}({\bf{u}})+{\kappa }^{k}{{\bf{I}}}_{{\rm{2}}}|$ at ${\bf{u}}={\hat{{\bf{u}}}}^{k}$, and ${c}^{k}$ is a constant independent of variables. Ignoring ${c}^{k}$, we write the minimization problem in $(k+{\rm{1}}){\rm{th}}$ iteration as

40 $$\begin{array}{rcl}\{{\hat{{\bf{u}}}}^{k+1},{\hat{{\bf{P}}}}^{k+1},{\hat{{\bf{E}}}}^{k+1}\} & = & \mathop{{\rm{\arg }}\,{\rm{\min }}}\limits_{{\bf{u}}\in {{\rm{C}}}^{{N}_{u}},{\bf{P}}\in {{\rm{C}}}^{ABC\times L},{\bf{E}}\in {{\rm{C}}}^{L\times L}}\frac{1}{2\sqrt{ABC}}\times \,({\rm{tr}}({{\bf{W}}}^{k}{T}_{bb}({\bf{u}}))+{\rm{tr}}({\bf{E}}))\\  &  & {\rm{subject}}\,{\rm{to}}[\begin{array}{cc}{T}_{bb}({\bf{u}}) & {\bf{P}}\\ {{\bf{P}}}^{{\rm{H}}} & {\bf{E}}\end{array}]\ge 0,{\Vert {{\bf{P}}}^{\bigstar}-{\bf{P}}\Vert }_{{\rm{F}}}\le \varepsilon \end{array},$$

which is also a disciplined convex optimization problem. Initialize ${\hat{{\bf{u}}}}^{0}={\bf{0}}$ and ${\kappa }^{0}=1$, then ${{\bf{W}}}^{0}={\bf{I}}$. The first iteration in Eq. (40) agrees with the ANM in Eq. (16). For the purpose of enhancing sparsity, *κ* should decrease gradually. The specific strategy is^18^

41 $${\kappa }^{k}=\{\begin{array}{ll}\min \,\{{\kappa }^{k-1}/2,\,{\lambda }_{\max }({T}_{bb}({\hat{{\bf{u}}}}^{k}))/\mathrm{10}\}, & k=1\\ {\kappa }^{k-1}/2, & {\rm{2}}\le k\le 10\\ {\kappa }^{10}, & k > 10\end{array},$$

where ${\lambda }_{\max }({T}_{bb}({\hat{{\bf{u}}}}^{k}))$ is the largest eigenvalue of ${T}_{bb}({\hat{{\bf{u}}}}^{k})$.

We define the weighted atomic norm of **P** as

42 $${\Vert {\bf{P}}\Vert }_{{{\rm{A}}}^{w}}=\mathop{{\rm{\inf }}}\limits_{\begin{array}{c}{\bf{d}}({t}_{1i},{t}_{2i},{t}_{{\rm{3}}i}){{\boldsymbol{\psi }}}_{i}\in {\rm{A}}\\ {s}_{i}\in {{\rm{R}}}^{+}\end{array}}\{\sum _{i}\frac{{s}_{i}}{w({t}_{1i},{t}_{2i},{t}_{{\rm{3}}i})}|{\bf{P}}=\sum _{i}{s}_{i}{\bf{d}}({t}_{1i},{t}_{2i},{t}_{{\rm{3}}i}){{\boldsymbol{\psi }}}_{i}\},$$

where $w({t}_{1i},{t}_{2i},{t}_{{\rm{3}}i})\ge {\rm{0}}$ is the weighting coefficient. When $w({t}_{1i},{t}_{2i},{t}_{{\rm{3}}i})\equiv {\rm{1}}$, ${\Vert {\bf{P}}\Vert }_{{{\rm{A}}}^{w}}={\Vert {\bf{P}}\Vert }_{{\rm{A}}}$. Denote by ${w}^{k}({t}_{1i},{t}_{2i},{t}_{3i})$ and ${\Vert {\bf{P}}\Vert }_{{{\rm{A}}}^{{w}^{k}}}$ the weighting coefficient and the weighted atomic norm corresponding to the results of the *k*th iteration. If

43 $${w}^{k}({t}_{1i},{t}_{2i},{t}_{3i})=\sqrt{\frac{ABC}{{\bf{d}}{({t}_{1i},{t}_{2i},{t}_{3i})}^{{\rm{H}}}{{\bf{W}}}^{k}{\bf{d}}({t}_{1i},{t}_{2i},{t}_{3i})}}$$

and ${T}_{bb}({\hat{{\bf{u}}}}^{k+1})$ admits a Vandermonde decomposition, then

44 $${\Vert {\bf{P}}\Vert }_{{{\rm{A}}}^{{w}^{k}}}=\mathop{{\rm{\min }}}\limits_{{\bf{u}}\in {{\rm{C}}}^{{N}_{u}},{\bf{E}}\in {{\rm{C}}}^{L\times L}}\frac{1}{2\sqrt{ABC}}({\rm{tr}}({{\bf{W}}}^{k}{T}_{bb}({\bf{u}}))+{\rm{tr}}({\bf{E}}))\,{\rm{subject}}\,{\rm{to}}\,[\begin{array}{cc}{T}_{bb}({\bf{u}}) & {\bf{P}}\\ {{\bf{P}}}^{{\rm{H}}} & {\bf{E}}\end{array}]\ge 0.$$

The proof can be found in Supplementary Note 5. Simultaneous Eqs (40) and (44) yield

45 $$\begin{array}{rcl}{\hat{{\bf{P}}}}^{k+1} & = & \mathop{{\rm{\arg }}\,{\rm{\min }}}\limits_{{\bf{P}}\in {{\rm{C}}}^{ABC\times L}}(\mathop{\min }\limits_{{\bf{u}}\in {{\rm{C}}}^{{N}_{u}},{\bf{E}}\in {{\rm{C}}}^{L\times L}}\frac{1}{2\sqrt{ABC}}\times \,({\rm{tr}}({{\bf{W}}}^{k}{T}_{bb}({\bf{u}}))+{\rm{tr}}({\bf{E}}))\,{\rm{subject}}\,{\rm{to}}[\begin{array}{cc}{T}_{bb}({\bf{u}}) & {\bf{P}}\\ {{\bf{P}}}^{{\rm{H}}} & {\bf{E}}\end{array}]\ge 0)\\  &  & \,{\rm{subject}}\,{\rm{to}}\,{\Vert {{\bf{P}}}^{\bigstar}-{\bf{P}}\Vert }_{{\rm{F}}}\le \varepsilon \\  & = & \mathop{{\rm{\arg }}\,{\rm{\min }}}\limits_{{\bf{P}}\in {{\rm{C}}}^{ABC\times L}}{\Vert {\bf{P}}\Vert }_{{{\rm{A}}}^{{w}^{k}}}\,{\rm{subject}}\,{\rm{to}}\,{\Vert {{\bf{P}}}^{\bigstar}-{\bf{P}}\Vert }_{{\rm{F}}}\le \varepsilon \end{array}$$

Apparently, **P** is reconstructed via minimizing its weighted atomic norm iteratively, and the weighting coefficient is updated in each iteration. Hence, the method can be named as IRANM. For sparse cuboid microphone arrays, ${{\bf{P}}}^{\bigstar}-{\bf{P}}$ in Eqs (37), (38), (40) and (45) becomes ${{\bf{P}}}_{{\rm{\Omega }}}^{\bigstar}-{{\bf{P}}}_{{\rm{\Omega }}}$.

### Implementation details

The simulations are conducted in Matlab R2014a on a PC with a Windows 10 system and a 2.2 GHz Intel(R) Core(TM) i5-5200U CPU. $A=B=C=7$ and ${\rm{\Delta }}x={\rm{\Delta }}y={\rm{\Delta }}z=0.035\,{\rm{m}}$. The number of snapshots is taken as 10. The independent and identically distributed complex Gaussian noise is utilized. The signal-to-noise ratio $(\mathrm{20lg}(\parallel {\bf{P}}{\parallel }_{{\rm{F}}}/\parallel {\bf{N}}{\parallel }_{{\rm{F}}}))$ is taken as 20 dB. In the ADMM based algorithm, $\tau$ is determined according to ref.^10^, $\rho$ is set to 1, and the iteration is terminated if the relative changes of **u** and **P** at two consecutive iterations, i.e., $\parallel {{\bf{u}}}^{q}-{{\bf{u}}}^{q-1}{\parallel }_{{\rm{2}}}/\parallel {{\bf{u}}}^{q-1}{\parallel }_{{\rm{2}}}$ and ${\Vert {{\bf{P}}}^{q}-{{\bf{P}}}^{q-1}\Vert }_{{\rm{F}}}/{\Vert {{\bf{P}}}^{q-1}\Vert }_{{\rm{F}}}$, both are less than 10^−3^ or the maximum number of iterations, set to 1000, is reached. Simulations of the ADMM based algorithm with another termination criterion relating to the primal and dual infeasibilities shown in Eqs (29) and (30) are also presented and discussed in Supplementary Note 6. In the MaPP method, the threshold is set as the maximal eigenvalue divided by 100, which means sources in 20 dB dynamic range are considered. When implementing IRANM, we terminate the iteration if the relative changes of the solution $\hat{{\bf{P}}}$ at two consecutive iterations, i.e., ${\Vert {\hat{{\bf{P}}}}^{k}-{\hat{{\bf{P}}}}^{k-1}\Vert }_{{\rm{F}}}/{\Vert {\hat{{\bf{P}}}}^{k-1}\Vert }_{{\rm{F}}}$, is less than 10^−3^ or the maximum number of iterations, set to 20, is reached.

## Supplementary information

Supplementary information

## Data Availability

Datasets generated and analyzed in the current study are available from the corresponding author on reasonable request.
