# Supplementary material for: A panoramic continuous compressive beamformer with cuboid microphone arrays
Source: Sci Rep. 2019 Aug 19;9:12073. doi: 10.1038/s41598-019-47845-7 (PMC6700166; doi:10.1038/s41598-019-47845-7)
Supplement: Supplementary file 1 — Supplementary information [file 41598_2019_47845_MOESM1_ESM.docx]

**Supplementary information**

A panoramic continuous compressive beamformer with cuboid microphone arrays

Yang Yang^1^, Zhigang Chu^2*^, Yong-Xin Yang^2^, Zhongming Xu^2^ & Yongxiang Zhang^2^

1. Faculty of Vehicle Engineering, Chongqing Industry Polytechnic College, Chongqing 401120, China.
2. State Key Laboratory of Mechanical Transmissions, Chongqing University, Chongqing 400044, China.

**Supplementary Note 1: An example to demonstrate advantages of multiple-snapshot data model over single-snapshot one**

*Parameter setup of sources*: Assume six sources. Their DOAs are (45°, 90°), (45°, 110°), (110°, 180°), (120°, 180°), (135°, 270°) and (155°, 290°). Their root mean square strengths are 100 dB, 98 dB, 96 dB, 94 dB, 92 dB and 90 dB. The frequency of emitted signal is 4000 Hz. The minimum separation among these sources is 0.06.

*Results*:


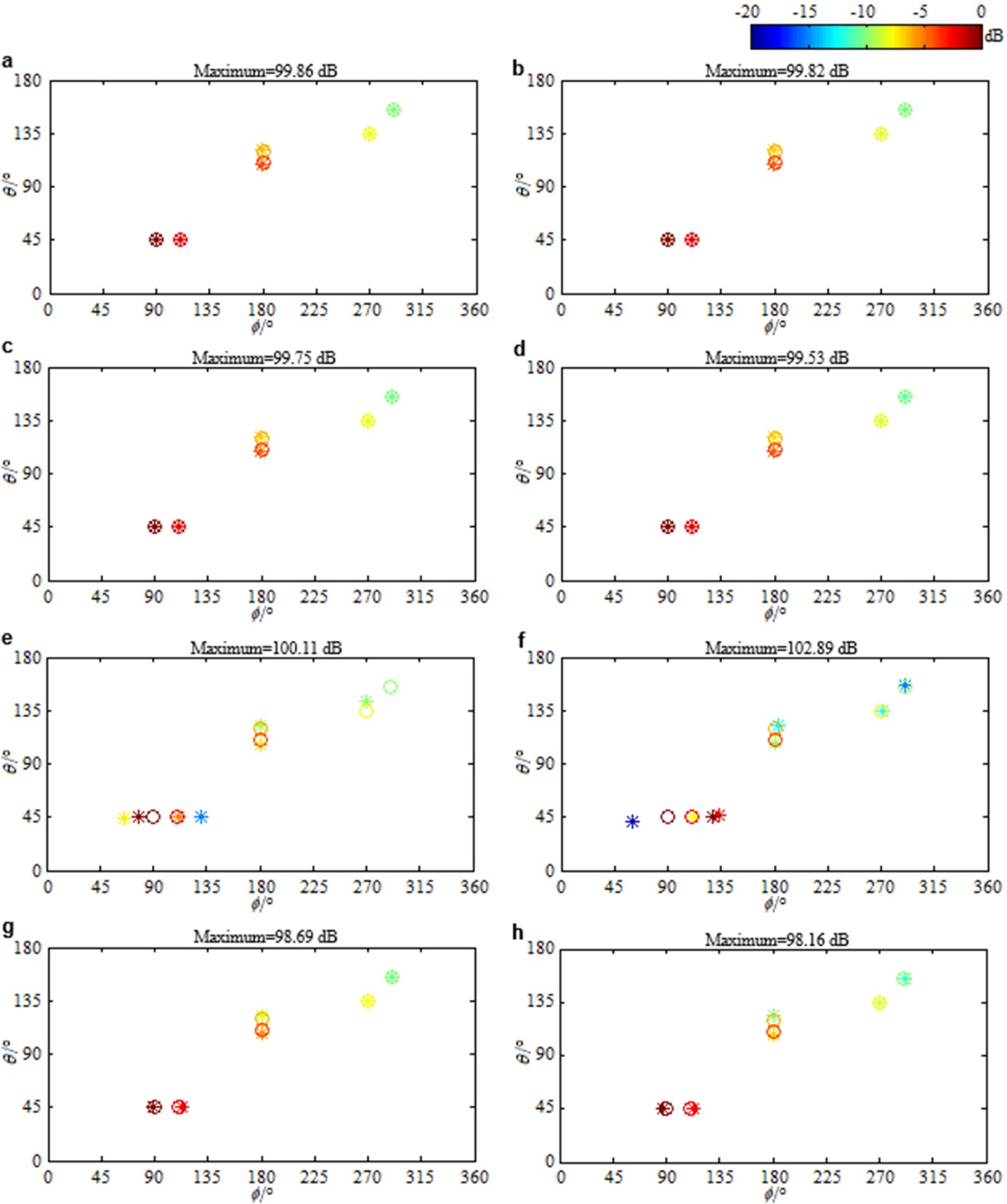


Fig. 1 Reconstructed source distributions. The number of snapshots is **a**-**d** 10 and **e**-**h** 1. The positive semidefinite programming equivalent to the ANM is solved by **a**, **b**, **e**, **f** the SDPT3 solver in CVX toolbox and **c**, **d**, **g**, **h** our ADMM based algorithm. **a**, **c**, **e**, **g** The standard uniform and **b**, **d**, **f**, **h** the sparse cuboid microphone array are utilized. In each map, the reconstructed (*) and the true (○) outputs are scaled to dB via referring to their respective maximum, and at the same time, referring to 2×10^-5^ Pa, the reconstructed maximum is labeled on the top.

*Discussion*: As shown in Fig. 1a-d, the beamformer can estimate the DOAs and quantify the strengths of these sources accurately when the multiple-snapshot data model is utilized. In contrast, as shown in Fig. 1e-h, it fails to obtain accurate results when the single-snapshot data model is utilized. This again demonstrates that the beamformer with the multiple-snapshot data model has stronger denoising capability, higher DOA estimation and strength quantification accuracy and enhanced resolution than the one with the single-snapshot data model.

**Supplementary Note 2: Convergence test of our ADMM based algorithm under different conditions**

Table 1 Specification of conditions corresponding to Fig. 2a-f.

| Subfigure | Condition | | | |
| --- | --- | --- | --- | --- |
|  | Number of sources | DOAs of sources | Frequency /Hz | SNR /dB |
| Fig. 2a | 5 | (30°, 100°), (45°, 120°), (70°, 150°), (110°, 200°), (135°, 250°) | 4000 | 20 |
| Fig. 2b | 4 | (20°, 70°), (60°, 100°), (80°, 130°), (120°, 210°) | 4000 | 20 |
| Fig. 2c | 6 | (45°, 90°), (45°, 120°), (90°, 180°), (120°, 180°), (135°, 270°), (155°, 290°) | 3000 | 20 |
| Fig. 2d | 6 | (45°, 90°), (45°, 120°), (90°, 180°), (120°, 180°), (135°, 270°), (155°, 290°) | 2000 | 20 |
| Fig. 2e | 6 | (45°, 90°), (45°, 120°), (90°, 180°), (120°, 180°), (135°, 270°), (155°, 290°) | 4000 | 10 |
| Fig. 2f | 6 | (45°, 90°), (45°, 120°), (90°, 180°), (120°, 180°), (135°, 270°), (155°, 290°) | 4000 | 30 |


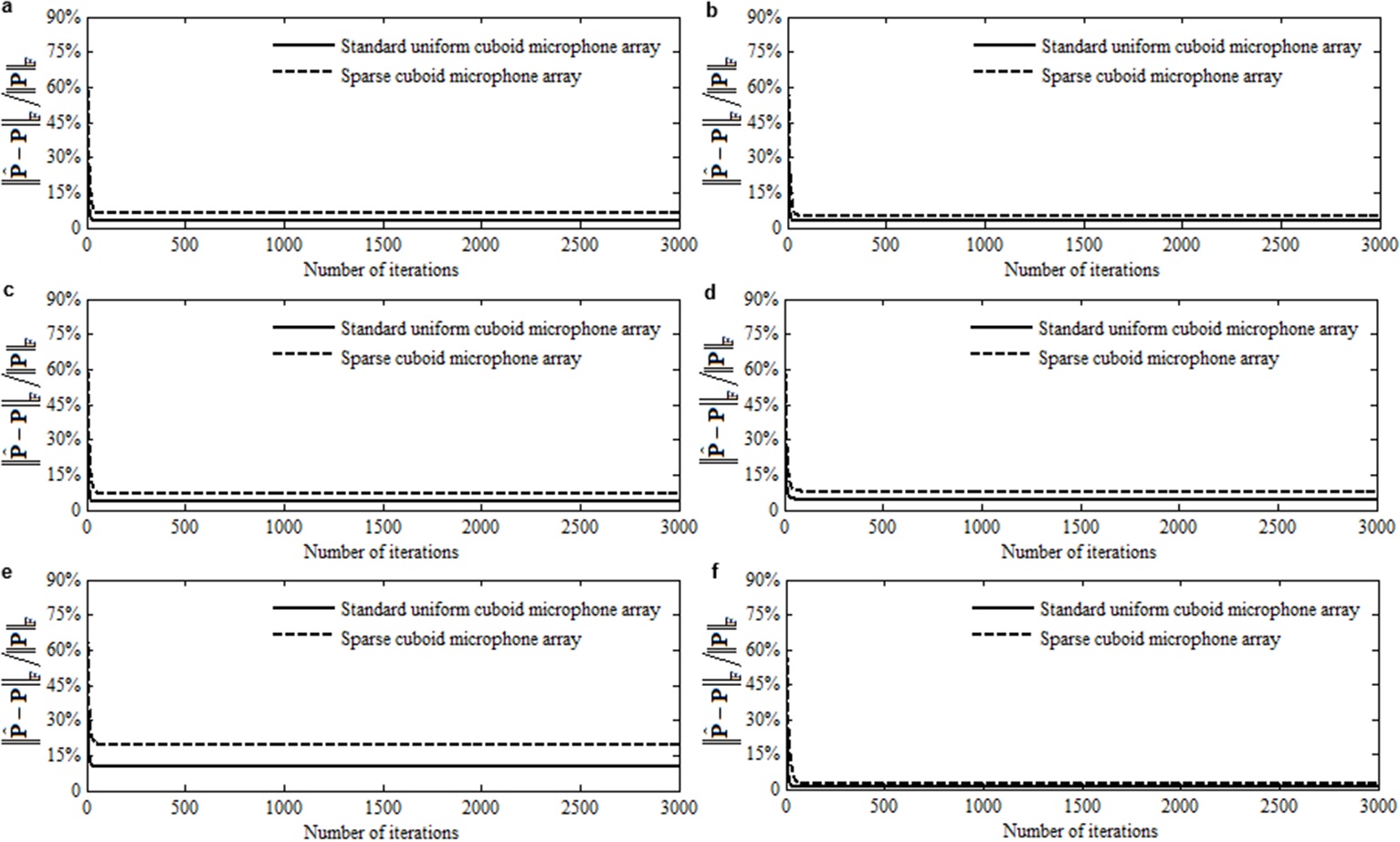


Fig. 2 Curves of $\left\|\hat{\mathbf{P}}-\mathbf{P}\right\|_{\text{F}}/\left\|\mathbf{P}\right\|_{\text{F}}$ vs. number of iterations under different conditions.

**Supplementary Note 3: Proof of the Proposition shown by Eqs. (13)-(15) in “Positive semidefinite programming to solve ANM”**

Two methods are given.

1) **Method One**

Let $\mathbf{P}\text{=}\sum_{i} s_{i}\mathbf{d}\left(t_{1i},t_{2i},t_{3i}\right)\mathbf{\psi}_{i}$, $T_{bb}\left(\mathbf{u}\right)=\left(1/\sqrt{ABC}\right)\sum_{i} s_{i}\mathbf{d}\left(t_{1i},t_{2i},t_{3i}\right)\mathbf{d}\left(t_{1i},t_{2i},t_{3i}\right)^{\text{H}}$ and $\mathbf{E}=\sqrt{ABC}\sum_{i} s_{i}\mathbf{\psi}_{i}^{\text{H}}\mathbf{\psi}_{i}$, then

. (1)

This suggests that $T_{bb}\left(\mathbf{u}\right)=\left(1/\sqrt{ABC}\right)\sum_{i} s_{i}\mathbf{d}\left(t_{1i},t_{2i},t_{3i}\right)\mathbf{d}\left(t_{1i},t_{2i},t_{3i}\right)^{\text{H}}$ and $\mathbf{E}=\sqrt{ABC}\sum_{i} s_{i}\mathbf{\psi}_{i}^{\text{H}}\mathbf{\psi}_{i}$ are the feasible solutions of the problem shown by Eq. (13) in the manuscript. Therefore,

. (2)

Since Inequation (2) holds for any decomposition of $\mathbf{P}$, we conclude that . On the other hand, if the Vandermonde decomposition shown by Eq. (15) in the manuscript holds, $\mathbf{P}$ falls within the column space of $T_{bb}\left(\hat{\mathbf{u}}\right)$, namely that $\mathbf{P}=\mathbf{V}\tilde{\mathbf{S}}$ with . Introduce a positive semidefinite matrix that satisfies $\hat{\mathbf{E}}=\tilde{\mathbf{S}}^{\text{H}}\mathbf{F}\tilde{\mathbf{S}}$, then

$\left[\begin{matrix} T_{bb}\left(\hat{\mathbf{u}}\right) & \mathbf{P} \\ \mathbf{P}^{\text{H}} & \hat{\mathbf{E}} \end{matrix}\right]\text{=}\left[\begin{matrix} \mathbf{V} & \mathbf{0} \\ \mathbf{0} & \tilde{\mathbf{S}}^{\text{H}} \end{matrix}\right]\left[\begin{matrix} \mathbf{\Sigma} & \mathbf{I} \\ \mathbf{I} & \mathbf{F} \end{matrix}\right]\left[\begin{matrix} \mathbf{V}^{\text{H}} & \mathbf{0} \\ \mathbf{0} & \tilde{\mathbf{S}} \end{matrix}\right]\underline{\succ}0$, (3)

where is an identity matrix. Inequation (3) yields $\left[\begin{matrix} \mathbf{\Sigma} & \mathbf{I} \\ \mathbf{I} & \mathbf{F} \end{matrix}\right]\underline{\succ}0$ and then $\mathbf{F}\underline{\succ}\mathbf{\Sigma}^{-1}$ by the Schur complement condition^26^. Now observe

$\text{tr}\left(\hat{\mathbf{E}}\right)=\text{tr}\left(\tilde{\mathbf{S}}^{\text{H}}\mathbf{F}\tilde{\mathbf{S}}\right)\geq\text{tr}\left(\tilde{\mathbf{S}}^{\text{H}}\mathbf{\Sigma}^{-1}\tilde{\mathbf{S}}\right)=\sum_{i} \sigma_{i}^{-1}s_{i}^{2}$. (4)

This implies

, (5)

where the second $\geq$ comes from the inequality of the arithmetic and geometric means and the third $\geq$ is due to the definition of atomic norm. Ultimately, .

2) **Method Two**

If $T_{bb}\left(\mathbf{u}\right)$ admits a Vandermonde decomposition, $\mathbf{P}$ falls within the column space of $T_{bb}\left(\mathbf{u}\right)$, namely that $\mathbf{P}=\mathbf{V}\tilde{\mathbf{S}}$ with . Then,

$\left[\begin{matrix} T_{bb}\left(\mathbf{u}\right) & \mathbf{P} \\ \mathbf{P}^{\text{H}} & \tilde{\mathbf{S}}^{\text{H}}\mathbf{\Sigma}^{-1}\tilde{\mathbf{S}} \end{matrix}\right]=\left[\begin{matrix} \mathbf{V\Sigma}^{\frac{1}{2}} \\ \left(\mathbf{\Sigma}^{-\frac{1}{2}}\tilde{\mathbf{S}}\right)^{\text{H}} \end{matrix}\right]\left[\begin{matrix} \mathbf{V\Sigma}^{\frac{1}{2}} \\ \left(\mathbf{\Sigma}^{-\frac{1}{2}}\tilde{\mathbf{S}}\right)^{\text{H}} \end{matrix}\right]^{\text{H}}\underline{\succ}0$, (6)

which yields $\tilde{\mathbf{S}}^{\text{H}}\mathbf{\Sigma}^{-1}\tilde{\mathbf{S}}\underline{\succ}\mathbf{P}^{\text{H}}T_{bb}\left(\mathbf{u}\right)^{-1}\mathbf{P}$ by the Schur complement condition. It follows that

. (7)

Hence,

, (8)

where the six equalities are due to Eqs. (13) and (14) in the manuscript, the Schur complement condition, Eq. (15) in the manuscript, Eq. (7), the inequality of the arithmetic and geometric means, and the definition of atomic norm, in turn.

**Supplementary Note 4: Derivation of Eqs. (23)-(26) in “ADMM to solve positive semidefinite programming”**

For a given matrix $\mathbf{A}$, $\left\|\mathbf{A}\right\|_{\text{F}}^{2}=\text{tr}\left(\mathbf{A}^{\text{H}}\mathbf{A}\right)$ holds. If $\mathbf{A}$ is Hermitian, $\left\|\mathbf{A}\right\|_{\text{F}}^{2}=\text{tr}\left(\mathbf{A}^{2}\right)$. For two given matrices $\mathbf{A}$ and $\mathbf{B}$ with the same dimensions, $\left\langle\mathbf{A},\mathbf{B}\right\rangle=\text{tr}\left(\mathbf{B}^{\text{H}}\mathbf{A}\right)$ holds. If $\mathbf{B}$ is Hermitian, $\left\langle\mathbf{A},\mathbf{B}\right\rangle=\text{tr}\left(\mathbf{BA}\right)$. For two given matrices $\mathbf{A}$ and $\mathbf{B}$, if the number of rows in $\mathbf{A}$ equals the number of columns in $\mathbf{B}$ and the number of columns in $\mathbf{A}$ equals the number of rows in $\mathbf{B}$, $\text{tr}\left(\mathbf{AB}\right)=\text{tr}\left(\mathbf{BA}\right)$ holds. According to these properties, we have

, (1)

, (2)

and

. (3)

Simultaneous Eqs. (1)-(3) and the expression of in Eq. (18) in the manuscript yield

. (4)

According to the complex-valued matrix derivatives (Hjørungnes, A. *Complex-Valued Matrix Derivatives: With Applications in Signal Processing and Communications* (Cambridge Univ. Press, Cambridge, UK, 2011).), we have

$\begin{matrix} \frac{\partial\text{tr}\left(\mathbf{A}\right)}{\partial\mathbf{A}}=\mathbf{I}_{0},\text{ }\frac{\partial\text{tr}\left(\mathbf{AB}\right)}{\partial\mathbf{A}}=\frac{\partial\text{tr}\left(\mathbf{BA}\right)}{\partial\mathbf{A}}=\mathbf{B}^{\text{T}},\text{ }\frac{\partial\text{tr}\left(\mathbf{A}^{p}\right)}{\partial\mathbf{A}}=p\left(\mathbf{A}^{\text{T}}\right)^{p-1}, \\ \frac{\partial\text{tr}\left(\mathbf{A}^{\text{H}}\mathbf{B}\right)}{\partial\mathbf{A}}=\frac{\partial\text{tr}\left(\mathbf{BA}^{\text{H}}\right)}{\partial\mathbf{A}}=\mathbf{0},\text{ and }\frac{\partial\text{tr}\left(\mathbf{A}^{\text{H}}\mathbf{A}\right)}{\partial\mathbf{A}}=\frac{\partial\text{tr}\left(\mathbf{AA}^{\text{H}}\right)}{\partial\mathbf{A}}=\mathbf{A}^{\text{*}} \end{matrix}$, (5)

where $\mathbf{I}_{0}$ is the identity matrix that has the same dimension as $\mathbf{A}$, $p$ is an exponent, and denotes the conjugate operator. Then,

, (6)

, (7)

, (8)

and

. (9)

Equations (6)-(9) all equal 0 when obtains its minimum. It follows that Eqs. (23)-(26) in the manuscript hold.

**Supplementary Note 5: Proof of the Proposition shown by Eqs. (43) and (44) in “IRANM”**

If $T_{bb}\left(\mathbf{u}\right)$ admits a Vandermonde decomposition, then

$\begin{matrix} \text{tr}\left(\mathbf{W}^{k}T_{bb}\left(\mathbf{u}\right)\right)=\sum_{i} \sigma_{i}\text{tr}\left(\mathbf{W}^{k}\mathbf{d}\left(t_{1i},t_{2i},t_{3i}\right)\mathbf{d}\left(t_{1i},t_{2i},t_{3i}\right)^{\text{H}}\right) \\ =\sum_{i} \sigma_{i}\text{tr}\left(\mathbf{d}\left(t_{1i},t_{2i},t_{3i}\right)^{\text{H}}\mathbf{W}^{k}\mathbf{d}\left(t_{1i},t_{2i},t_{3i}\right)\right) \\ =\sum_{i} \sigma_{i}\mathbf{d}\left(t_{1i},t_{2i},t_{3i}\right)^{\text{H}}\mathbf{W}^{k}\mathbf{d}\left(t_{1i},t_{2i},t_{3i}\right) \end{matrix}$. (1)

Hence,

,(2)

where the six equalities are due to the Schur complement condition, the condition that $T_{bb}\left(\hat{\mathbf{u}}^{k+1}\right)$ admits a Vandermonde decomposition, Eq. (1) here and Eq. (7) in Supplementary Note 1, the inequality of the arithmetic and geometric means, Eq. (43) in the manuscript, and the definition of weighted atomic norm, in turn.

**Supplementary Note 6: Simulations of the ADMM based algorithm with another termination criterion**

*Parameter setup of sources*: same as in Fig. 1.

*Termination criterion of the ADMM based algorithm*: $\max\left\{\text{pri}_{-}\inf^{q},\text{dual}_{-}\inf^{q}\right\}<\delta$.

*Results*:


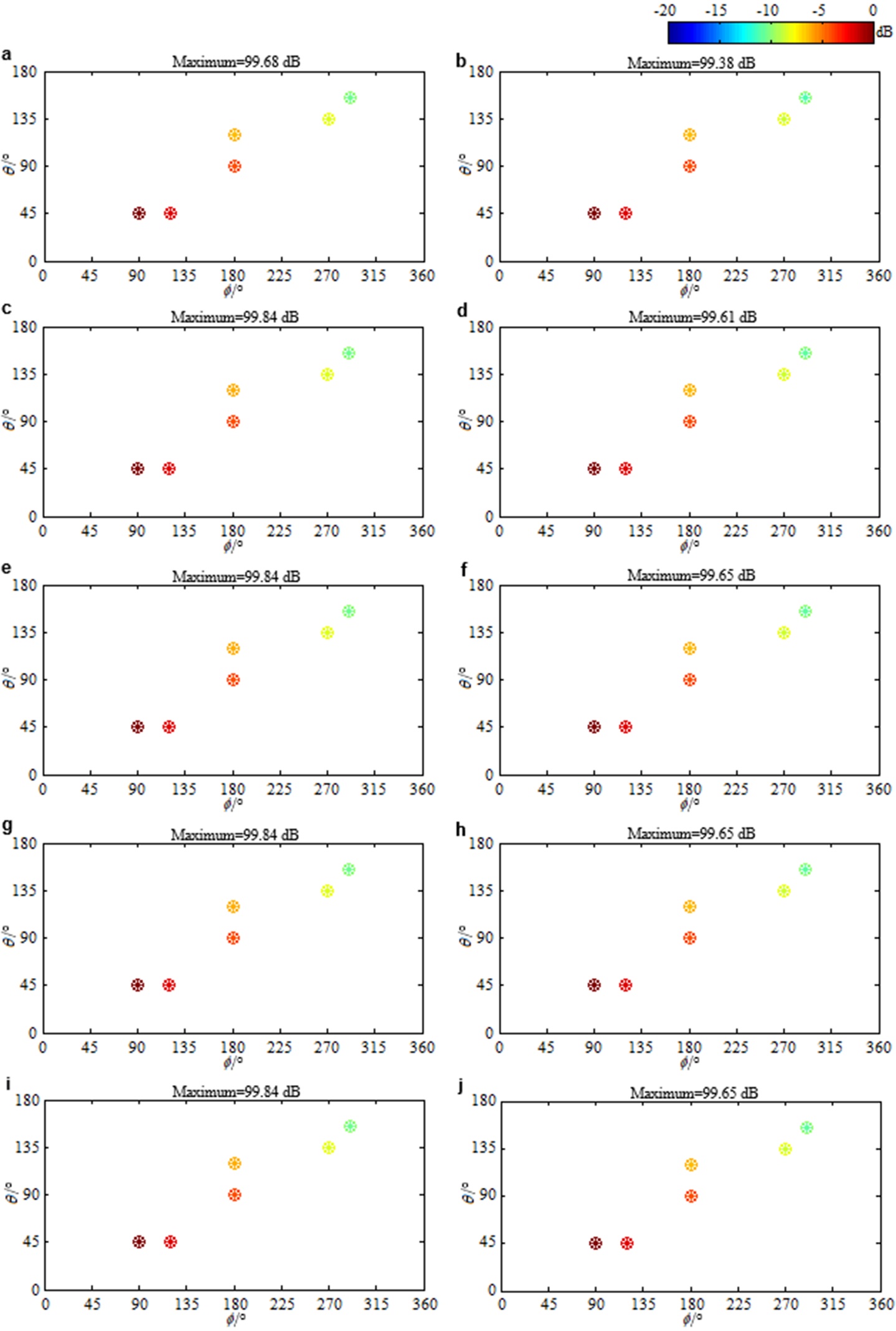


Fig. 3 Reconstructed source distributions. $\delta$ is **a**, **b** 10^-1^, **c**, **d** 10^-2^, **e**, **f** 10^-3^, **g**, **h** 10^-4^, **i**, **j** 10^-5^. **a**, **c**, **e**, **g**, **i** The standard uniform and **b**, **d**, **f**, **h**, **j** the sparse cuboid microphone array are utilized. In each map, the reconstructed (*) and the true (○) outputs are scaled to dB via referring to their respective maximum, and at the same time, referring to 2×10^-5^ Pa, the reconstructed maximum is labeled on the top.

Table 2 Microphone signal reconstruction error, DOA estimation error and source strength quantification error corresponding to Fig. 3, and needed iterative number and consuming time of ADMM based algorithm.

| Microphone array | $\delta$ | $\frac{\left\Vert\hat{\mathbf{P}}-\mathbf{P}\right\Vert_{\text{F}}}{\left\Vert\mathbf{P}\right\Vert_{\text{F}}}$ |  | $\frac{\left\Vert\hat{\mathbf{s}}_{rms}-\mathbf{s}_{rms}\right\Vert_{2}}{\left\Vert\mathbf{s}_{rms}\right\Vert_{2}}$ | Needed iterative number | Consuming time /s |
| --- | --- | --- | --- | --- | --- | --- |
| Standard uniform cuboid array with 343 microphones | 10^-1^ (Fig. 3a) | 5.54% | 0.06% | 5.11% | 19 | 91 |
|  | 10^-2^ (Fig. 3c) | 3.40% | 0.06% | 3.09% | 71 | 341 |
|  | 10^-3^ (Fig. 3e) | 3.39% | 0.06% | 3.07% | 271 | 1255 |
|  | 10^-4^ (Fig. 3g) | 3.38% | 0.06% | 3.07% | 491 | 2343 |
|  | 10^-5^ (Fig. 3i) | 3.38% | 0.06% | 3.07% | 719 | 3459 |
| Sparse cuboid array with 170 microphones | 10^-1^ (Fig. 3b) | 9.80% | 0.08% | 9.27% | 31 | 157 |
|  | 10^-2^ (Fig. 3d) | 6.93% | 0.05% | 6.63% | 50 | 250 |
|  | 10^-3^ (Fig. 3f) | 6.39% | 0.05% | 6.13% | 226 | 1099 |
|  | 10^-4^ (Fig. 3h) | 6.38% | 0.05% | 6.13% | 435 | 2125 |
|  | 10^-5^ (Fig. 3j) | 6.38% | 0.05% | 6.13% | 651 | 3175 |

*Discussion*:

- Figure 3 shows that when the ADMM based algorithm with $\max\left\{\text{pri}_{-}\inf^{q},\text{dual}_{-}\inf^{q}\right\}<\delta$ ($\delta$=10^-1^, 10^-2^, 10^-3^, 10^-4^ or 10^-5^) as termination criterion is utilized, the beamformer can estimate the DOAs and quantify the strengths of these sources.
- Table 2 shows that on one hand, when $\delta\geq10^{-2}$, a smaller $\delta$ means lower microphone signal reconstruction, DOA estimation and source strength quantification errors, while when $\delta\leq10^{-2}$, these errors almost keep unchanged as $\delta$ decreases. On the other hand, a smaller $\delta$ means more iterative number and consuming time. Therefore, $\delta=10^{-2}$ is a perfect choice.
- Comparing Fig. 3c, d and Table 2 here with Fig. 1g, h and Table 1 in the manuscript, we can see that the results presented in the manuscript are comparable to the ones here corresponding to $\delta=10^{-2}$. This means that the termination criterion used in the manuscript, i.e., the iteration is terminated if the relative changes of $\mathbf{u}$ and $\mathbf{P}$ at two consecutive iterations both are less than 10^-3^ or the maximum number of iterations, set to 1000, is reached, is reasonable. Besides, the termination criterion is more convenient to use.
- Table 2 shows that 719 and 651 iterations are needed even when $\delta=10^{-5}$. Therefore, it is enough to set the maximum number of iterations as 1000.
- Whether the standard uniform or the sparse cuboid microphone array is utilized, these rules stand up.
